# Transdermal rivastigmine for HIV-associated cognitive impairment: A randomized pilot study

**DOI:** 10.1371/journal.pone.0182547

**Published:** 2017-08-30

**Authors:** Jose A. Muñoz-Moreno, Anna Prats, José Moltó, Maite Garolera, Núria Pérez-Álvarez, Crisanto Díez-Quevedo, Cristina Miranda, Carmina R. Fumaz, Maria J. Ferrer, Bonaventura Clotet, on behalf of the TRIANT-TE Study Group

**Affiliations:** 1 Fundació Lluita contra la SIDA (FLS), Hospital Universitari Germans Trias i Pujol, Badalona, Catalonia, Spain; 2 Facultat de Psicologia i Ciències de l'Educació, Universitat Oberta de Catalunya (UOC), Barcelona, Catalonia, Spain; 3 Departament de Psiquiatria i Medicina Legal, Universitat Autònoma de Barcelona (UAB), Cerdanyola del Vallès, Catalonia, Spain; 4 Clinical Research Group for Brain, Cognition and Behavior, Consorci Sanitari Hospital de Terrassa, Terrassa, Catalonia, Spain; 5 Grup de Recerca Consolidat en Neuropsicologia, Universitat de Barcelona (UB), Barcelona, Catalonia, Spain; 6 Departament d'Estadística i Investigació Operativa, Universitat Politècnica de Catalunya (UPC), Barcelona, Catalonia, Spain; 7 Servei de Psiquiatria, Hospital Universitari Germans Trias i Pujol, Badalona, Catalonia, Spain; 8 Institut per la Recerca de la SIDA IrsiCaixa, Badalona, Catalonia, Spain; 9 Càtedra de la SIDA i Malalties Relacionades, Universitat de Vic—Universitat Central de Catalunya (UVic), Vic, Catalonia, Spain; Imperial College London, UNITED KINGDOM

## Abstract

**Objective:**

To assess the efficacy and safety of transdermal rivastigmine for the treatment of HIV-associated cognitive impairment.

**Methods:**

We recruited HIV-infected patients with cognitive impairment on stable antiretroviral therapy in a randomized controlled pilot trial with a 48-week follow-up. An additional assessment was held at 12 weeks. Participants received transdermal rivastigmine (9.5 mg daily), lithium (400 mg twice daily, titrated progressively), or remained in a control group (no new medication). The primary efficacy endpoint was change in a global cognitive score (NPZ-7). Secondary endpoints included change in specific cognitive measures, domains, and functional parameters. Safety covered the frequency of adverse events and changes in laboratory results.

**Results:**

Seventy-six subjects were screened, and 29 were finally enrolled. Better cognitive outcomes were observed in all groups, although there were no significant differences between the arms (mean NPZ-7 change [SD]): rivastigmine, 0.35 (0.14); lithium, 0.25 (0.40); control, 0.20 (0.44) (*p* = 0.78). The rivastigmine group showed the highest positive trend (mean NPZ-7 [SD], baseline vs week 48): rivastigmine, –0.47 (0.22) vs –0.11 (0.29), *p* = 0.06; lithium, –0.50 (0.40) vs –0.26 (0.21), *p* = 0.22; control, –0.52 (0.34) vs –0.32 (0.52), *p* = 0.44. The cognitive domains with the highest positive trends were information processing speed at week 12 and executive function at week 48 (rivastigmine vs control): information processing speed, 0.35 (0.64) vs –0.13 (0.25), *p* = 0.17, *d* = 0.96; and executive functioning, 0.73 (0.33) vs 0.03 (0.74), *p* = 0.09, *d* = 1.18. No relevant changes were observed regarding functional outcomes. A total of 12 (41%) individuals dropped out of the study: 2 (20%) were due to medication-related effects in the rivastigmine group and 4 (36%) in the lithium group. No severe adverse events were reported.

**Conclusions:**

The results from this small randomized trial indicate that transdermal rivastigmine did not provide significant cognitive benefits in people with HAND on stable antiretroviral therapy, even though positive trends were found in specific cognitive domains. Relevant tolerability issues were not observed.

## Introduction

Cognitive impairment has become a persistent complication in people with HIV infection, despite the use of combination antiretroviral therapy (cART). Reports show a prevalence of between 30% and 60% in people living with HIV during the recent cART era [1,2], and this impairment appears to have a negative effect on quality of life [3], daily functioning [4], and clinical outcomes (e.g., poor adherence to antiretroviral therapy and frequent virological failure) [5,6]. Adjuvant pharmacological therapies have been suggested for the management of HIV-associated neurocognitive disorders (HAND), although most trials investigating them have not proved clear benefits [7].

Rivastigmine is a cholinesterase inhibitor used to treat cognitive impairment, mainly in Alzheimer's and Parkinson's diseases [8]. Rivastigmine halts the action of the enzyme acetylcholinesterase, thus increasing levels of acetylcholine in the brain and enhancing the function of neural cells, particularly those involved in attention and working memory processes [9]. This drug also has beneficial non-cholinergic effects, including Aβ brain load reduction and neuroprotective and anti-inflammatory effects in vivo and in vitro [10,11]. Both animal and human studies have demonstrated that Aβ metabolism can be altered by HIV infection [12,13]. Oral rivastigmine was tested in a randomized study of patients with HAND [14]. The authors reported benefits in information processing speed, even though no significant improvement in global cognitive functioning was established. Adverse effects were common (76%). Transdermal rivastigmine has a better tolerability profile than the oral formulation [8], although it has not been assessed in the field of HIV infection. To date, the only compound that has proven beneficial is lithium; however, its benefits appear to be variable, and the incidence of adverse events, particularly hypothyroidism, diabetes insipidus, and lithium toxicity, is high [15,16].

We carried out a randomized controlled pilot trial with a 48-week follow-up, in which patients received transdermal rivastigmine, lithium, or did not start new medication. We assessed the safety and efficacy profile, considering change in cognitive functioning as the main efficacy endpoint, and changes in specific measures, domains, and functional outcomes, as secondary efficacy endpoints.

## Material and methods

### Participants

The TRI-ANTiretroviral and Adjuvant ThErapies for HIV-associated cognitive impairment (TRIANT-TE) trial was designed to investigate the safety and efficacy of transdermal rivastigmine as treatment for cognitive impairment in HIV-infected patients. The study was performed in the HIV Clinical Unit of the Germans Trias i Pujol University Hospital, Badalona, Catalonia, Spain. Patients suspected of having HIV-associated cognitive impairment were invited to participate in the study. The inclusion criteria were age 20 to 75 years, confirmed HIV infection, stable antiretroviral therapy (previous 6 months), confirmed undetectable plasma viral load, confirmed cognitive impairment (see Assessments section), Catalan or Spanish as the native language, full understanding of the objectives of the study, and written informed consent. The exclusion criteria were pregnancy, therapy contraindicated with the study medications, and complications that prevented the patient from starting the study drugs. Potential confounding comorbidities for cognitive impairment were also considered exclusion criteria and were based on the proposal of the Frascati group [17]. Those comorbidities included current or previous CNS-related disease, current or previous psychiatric disorder, current use of psychopharmacological medication, use of illicit drugs, and coinfection with hepatitis C virus. The screening period was from May 2011 to May 2013.

### Study design

We developed a randomized controlled pilot trial, whose primary and secondary endpoints were established for a 48-week follow-up. An additional assessment was performed at week 12. Patients were randomized 1:1:1 to transdermal rivastigmine, lithium, or no new medication. This assignment included stratification according to the antiretroviral CNS penetration-effectiveness (CPE) score [18]. The score was balanced at baseline to avoid undesirable antiretroviral effects on CNS functioning. We calculated the mean score to be 8 in the population attended in the center, and, therefore, we established 2 randomization tables depending on whether the score was <8 or ≥8. After randomization, participants were assigned to 1 of the following study arms; the rivastigmine group (RG), which comprised patients who initiated therapy with transdermal rivastigmine (*Prometax*^*®*^, *Novartis*, *AG*) and in which dosing was started at 4.6 mg daily and increased to 9.5 mg daily at week 4; or the lithium group (LG), in which patients initiated therapy with oral lithium (*Plenur*^*®*^, *Faes Farma*, *SA*) and in which dosing was started at 400 mg twice daily before being titrated progressively to ensure plasma drug concentrations of between 0.4 and 0.8 mEq/l. An appointment was made for 2 weeks and 4 weeks to monitor this dosing schedule. The control group (CG) comprised participants who did not initiate a new treatment. Adherence to both antiretroviral therapy and study medication were monitored using the Self-Reported Adherence (SERAD) questionnaire [19]. Despite the non-blinded character of the study, neuropsychologists were unaware of the specific participation in each trial arm.

The study was managed by the Lluita contra la SIDA Foundation (http://www.flsida.org). All study procedures were conducted in accordance with the 1964 Declaration of Helsinki (fourth revision, 1996) and Good Clinical Practice guidelines. The study was approved by the Research Ethics Committee of Germans Trias i Pujol University Hospital (*project code*: *EO-07-039*). All patients gave their informed consent before enrolment. The trial is registered at ClinicalTrials.gov (*NCT01348282*).

### Assessments

Patients underwent a comprehensive battery of neuropsychological tests that evaluated 7 cognitive domains. The tests applied and the domains assessed were the following: the Letter-Number and Digit tests of the Wechsler Adult Intelligence Scale—Version III (WAIS-III) [20], for attention/working memory; the Trail Making Test—Part A (TMT-A) [21] and the Symbol Digit Modalities Test (SDMT) [22], for information processing speed; the California Verbal Learning Test—Version II (CVLT-II) [23], for verbal memory and learning; the Trail Making Test—Part B (TMT-B) [21], the Stroop Test [24], the Wisconsin Card Sorting Test (WCST) [25], and the Tower of London Test (TOL) [26], for executive functioning; the Controlled Oral Word Association Test (COWAT) [27] and the Animals Test [28], for verbal fluency; and the Grooved Pegboard Test (GPT) [29], for motor function. The Vocabulary Test of the WAIS-III was used to monitor premorbid intelligence. Cognitive impairment was defined as performing ≥1 standard deviation below the normative mean in ≥2 of the cognitive domains assessed, as extensively reported in other studies in the field. Standardized z scores were used for all comparisons. They were obtained after adjusting the raw scores according to available normative data in Catalan/Spanish and English, which covered principally age, gender, and educational level [30–34]. The battery used for screening was the same as that used in the 12- and 48-week study assessments. Cognitive complaints were recorded based on the European AIDS Clinical Society guidelines [35]. HAND were determined based on the Frascati criteria [17]. Functional outcomes were assessed by evaluating daily living, quality of life, and emotional status. Daily living was evaluated using a version of the Instrumental Activities of Daily Living (IADL) questionnaire in Catalan/Spanish, which covered 12 functional dimensions and included a score measuring the total number of impaired areas [4]. Quality of life was recorded using a reduced version of the Medical Outcomes Study HIV Health Survey (MOS-HIV) questionnaire [36], which covered 4 specific quality of life dimensions. Emotional status was evaluated in terms of depression and anxiety symptoms, both of which were assessed using the Hospital Anxiety and Depression Scale (HADS) [37]. Satisfaction was assessed based on 3 variables—effort to take the new medication, ease of management of the new medication, and general satisfaction with the treatment—and scored using 0–10–point Likert scales.

### Statistical analyses

The data analyses were based mainly on prospective comparisons of primary and secondary outcomes between baseline and 48-week study visits. They were applied with 2 groups (medication group vs control group) or all 3 groups (RG, LG, and CG) based on descriptive results or the study endpoints. The primary efficacy endpoint was the change in neurocognitive functioning from baseline to week 48 measured using the NPZ-7 score. The endpoint consisted of a global composite measure comprising the mean of 7 neurocognitive measures, specifically the letter-number total score (WAIS-III), TMT-A total time, long-term free recall (CVLT-II), total A list (CVLT-II), TMT-B total time, COWAT total score, and GPT non-dominant hand score. This score combination was selected because it has been shown to provide optimal sensitivity and specificity for the detection of HIV-associated cognitive impairment [38]. The secondary endpoints comprised changes in specific cognitive measures and domains, daily living, quality of life, and emotional variables, as well as satisfaction with the new treatment. The safety analysis took account of changes in the frequency, type, and severity of adverse events. Abnormal results in laboratory tests and changes over time in vital signs and symptoms were also assessed. Additional comparisons included the assessment at week 12. Standardized z scores were used for all comparisons with regard to cognitive endpoints. They were obtained from local normative data when available, and were based principally on age, gender, and educational level. Daily living, quality of life, and emotional outcomes were treated as numerical outcomes from raw scores. Satisfaction levels were numerical and were compared between medication arms. The statistical tests were applied according to the type of variable and included the *t* test, chi-square test, and ANCOVA. All comparisons were univariate and 2-tailed. Statistical significance was set at *p*<0.05. Supplementary analyses with ANCOVA were performed to study differences between the groups after adjustment for the variables that were significantly unbalanced at baseline. Cohen's effect size tests were performed to quantify the magnitude of the differences found. Values were considered small when scores were less than 0.40, medium when they ranged between 0.40 and 0.75, and large when they were over 0.75. All statistical analyses were performed using SPSS Statistics^®^, version 15 (SPSS Inc, Chicago, Illinois, USA).

## Results

### Baseline characteristics

**Fig 1:**
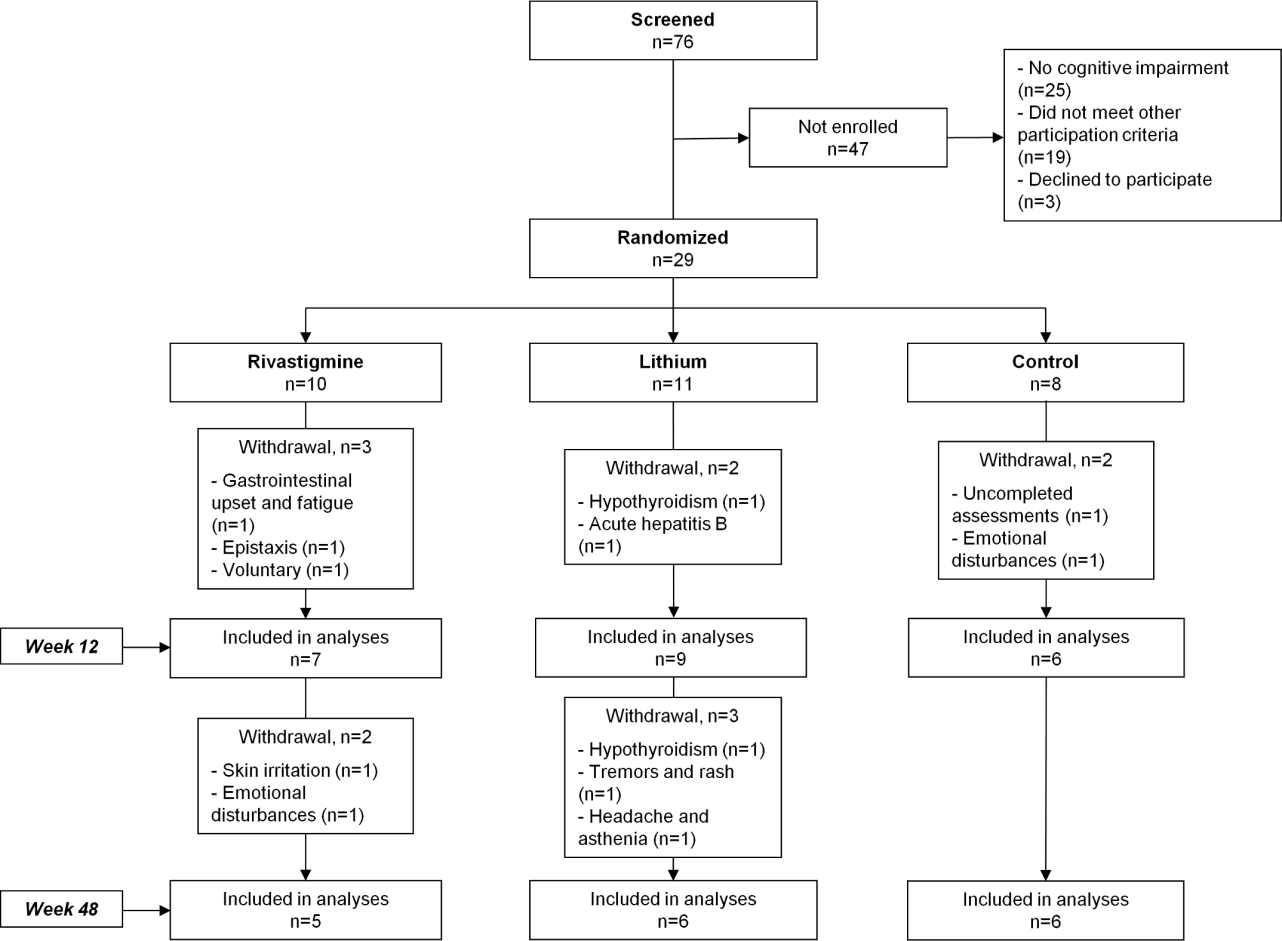
Flow diagram of trial participation.

A total of 76 HIV-infected patients were screened. Twenty-five (32%) did not present cognitive impairment, 19 (25%) did not meet the other study criteria, and 3 (4%) opted not to participate (**Fig 1**). Thus, 29 individuals were randomized. By arm, 10 patients were allocated to the RG, 11 to the LG, and 8 to the CG.

Most patients were men (79%) and men who had sex with men (MSM) (55%), with a mean (standard deviation) age of 44 (5) years. Mean time since HIV diagnosis was 12 (7) years, and patients presented a mean CD4 count of 578 (217) cells/μL and a nadir CD4 count of 222 (121) cells/μL. Time since HIV diagnosis was not distributed equally between the groups (RG, 17 [7]; LG, 8 [4]; CG, 12 [7]; *p* = 0.02) (**Table 1**). This variable was included in the supplementary ANCOVAs.

**Table 1:** Baseline characteristics of study participants.

		Rivastigmine (n = 10)	Lithium (n = 11)	Control (n = 8)	*p* Value
**Age, y, mean (SD)**		45 (7)	43 (5)	45 (3)	0.592
**Male, n (%)**		7 (70)	9 (81)	7 (87)	0.640
**Years of education, mean (SD)**		11 (4)	13 (5)	12 (4)	0.497
**Route of transmission, n (%)**					0.255
	**Injecting drug user**	2 (20)	2 (18)	2 (25)	
	**Heterosexual**	4 (40)	2 (18)	0 (0)	
	**MSM**	4 (40)	6 (55)	6 (75)	
	**Other**	0 (0)	1 (9)	0 (0)	
**Time since HIV diagnosis, y, mean (SD)**		17 (7)	8 (4)	12 (7)	0.020*
**Months on current ARV regimen, mean (SD)**		34 (30)	31 (24)	36 (22)	0.918
**ARV regimen, n (%)**					0.661
	**NNRTI-based**	2 (20)	5 (45)	4 (50)	
	**PI-based**	6 (60)	5 (45)	3 (38)	
	**II-based**	2 (20)	1 (10)	1 (12)	
**CPE score, mean (SD)** ^***a***^		7.4 (1.5)	7.0 (2.6)	7.8 (1.1)	0.633
**CD4 cell count, mean (SD)**		591 (211)	649 (206)	463 (218)	0.180
**Nadir CD4 cell count, mean (SD)**		180 (126)	280 (100)	194 (123)	0.124
**Undetectable plasma viral load, n (%)** ^***b***^		10 (100)	11 (100)	8 (100)	-
**Highest plasma viral load, mean (SD)**		228,525 (305,909)	379,200 (481,250)	100,666 (106,281)	0.298
**Depression symptoms, mean (SD)** ^***c***^		8 (5)	6 (4)	7 (4)	0.789
**Anxiety symptoms, mean (SD)** ^***c***^		9 (4)	10 (3)	8 (4)	0.765
**Cognitive complaints, n (%)** ^***d***^		6 (60)	10 (91)	7 (87)	0.184
**NPZ-7, mean (SD)**		–0.56 (0.59)	–0.29 (0.95)	–0.58 (0.35)	0.584
**HAND, n (%)** ^***e***^					0.196
	**ANI**	2 (20)	0 (0)	1 (12)	
	**MND**	8 (80)	11 (100)	7 (88)	
	**HAD**	0 (0)	0 (0)	0 (0)	

Abbreviations: ANI, asymptomatic neurocognitive impairment; ARV, antiretroviral; CPE, central nervous system penetration-effectiveness; HAD, HIV-associated dementia; HAND, HIV-associated neurocognitive disorder; II, integrase inhibitor; MND, mild neurocognitive disorder; MSM, men who had sex with men; NNRTI, non-nucleoside reverse transcriptase inhibitor; PI, protease inhibitor.

^a^ Based on the proposal by Letendre et al (2010).^18^

^b^ Detection limit of ≤40 copies/mL.

^c^ Assessed using the Hospital Anxiety-Depression Scale (HADS).^39^

^d^ Based on the proposal by the European AIDS Clinical Society (EACS).^36^

^e^ According to the Frascati criteria (2007).^17^

* p<0.05.

No patients changed their cART regimen during the study, and there were no relevant changes in viral load. Adherence to study medication at week 48 was ≥90% for all patients in the RG; 1 patient in the LG reported <90% adherence. The mean percentages were 100% (0%) and 91% (19%), respectively. Median lithium drug levels were in the expected therapeutic range at 12 weeks (0.5 [0.4–0.6] mEq/l) and at 48 weeks (0.5 [0.3–0.8] mEq/l).

### Efficacy outcomes

At week 12, no differences were found in the primary study endpoint. All groups showed better cognitive outcomes, although none reached statistical significance (mean [SD] NPZ-7 change): RG, 0.27 (0.38); LG, 0.10 (0.21); CG, 0.15 (0.53); *p* = 0.66. The most pronounced trend towards improvement was observed in the RG (mean [SD] NPZ-7, baseline vs wk12): RG, –0.38 (0.28) vs –0.11 (0.30), *p* = 0.10; LG, –0.26 (0.28) vs –0.16 (0.23), *p* = 0.43; CG, –0.52 (0.34) vs –0.39 (0.57), *p* = 0.63. The WAIS-III digit span backward score (*attention/working memory*) revealed significant differences, with a large effect size, showing better functioning in the RG than in the CG (RG vs CG): 0.10 (0.26) vs –0.48 (0.37), *p*<0.01, *d* = 1.84. The domain that improved most was information processing speed in the RG (RG vs CG): 0.35 (0.64) vs –0.13 (0.25), *p* = 0.17, *d* = 0.96. With regard to daily living and quality of life outcomes, no differences were observed in any areas. Symptoms of depression and anxiety were less frequent in both the RG and the LG, particularly in the LG, albeit without reaching statistical significance: RG, –1.57 (3.45); LG, –3.37 (3.11); CG, –0.20 (3.27); *p* = 0.24; RG, –1.00 (2.70); LG, –1.75 (4.74); CG, 2.40 (4.82); *p* = 0.22, respectively.

**Fig 2:**
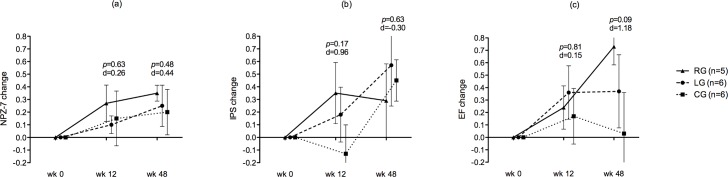
Changes in NPZ-7 and in information processing speed and executive functioning domains. (a) Mean change in NPZ-7. (b) Mean change in information processing speed. (c) Mean change in executive functioning. Values are expressed as z-score means, bars as standard errors. P values and d sizes are provided for rivastigmine vs control group comparisons. Abbreviations: CG, control group; EF, executive functioning; IPS, information processing speed; LG, lithium group; RG, rivastigmine group.

At week 48, cognitive outcomes were still better in all groups, although significant differences were still not found for the primary endpoint (mean [SD] NPZ-7 change): RG, 0.35 (0.14); LG, 0.25 (0.40); CG, 0.20 (0.44); *p* = 0.78 (***Table 2***). The most pronounced trend towards benefits was observed for the RG (mean [SD] NPZ-7, baseline vs week 48): RG, –0.47 (0.22) vs –0.11 (0.29), *p* = 0.06; LG, –0.50 (0.40) vs –0.26 (0.21), *p* = 0.22; CG, –0.52 (0.34) vs –0.32 (0.52), *p* = 0.44. The adjusted analyses with the supplementary ANCOVA, which were corrected for time since HIV diagnosis, confirmed the unadjusted results. The TOL total moves measure (*executive functioning*) revealed a trend towards benefits in the RG and a significantly better score in the LG when both were compared with the CG, with large effect sizes in both cases: RG vs CG, 0.70 (0.73) vs –0.61 (1.28), *p* = 0.07, *d* = 1.22; LG vs CG, 1.05 (0.81) vs –0.61 (1.28), *p =* 0.02, *d* = 1.55. In terms of domains, executive functioning revealed a slightly clearer trend towards improvement in the RG, with a large effect size: RG vs CG: 0.73 (0.33) vs 0.03 (0.74), *p* = 0.09, *d* = 1.18 (**Fig 2**). No relevant associations were found when other potential explicative variables (e.g., education level, age, or time since HIV diagnosis) were included in the analyses. Regarding daily living, quality of life, and emotional status, significant differences were only found for a single measure of daily activities (*cooking*), with a better score in the RG than in the CG: RG vs CG: –0.40 (0.54) vs 0.33 (0.81), *p* = 0.04, *d* = –1.39 (**Table 3**). Satisfaction levels did not differ between the medication groups and were fairly positive overall (RG vs LG): effort, 1.6 (3.04) vs 3.33 (3.88), *p* = 0.43, *d* = –0.49; ease, 6.8 (3.27) vs 7.83 (1.72), *p* = 0.51, *d* = –0.41; satisfaction, 7 (1) vs 7 (2.52), *p* = 1.00, *d* = 0.00.

**Table 2:** Change in cognitive measures at week 48.

		Rivastigmine (n = 5)	Lithium (n = 6)	Control (n = 6)	*p* Value ^*a*^^;^ ^*b*^	*d* Size ^*a*^^;^ ^*b*^
**Attention/working memory**						
	**Digit span forward (WAIS-III)**	0.28 (0.38)	0.11 (0.68)	0.58 (0.68)	0.404; 0.267	–0.53; –0.69
	**Digit span backward (WAIS-III)**	–0.32 (0.43)	–0.01 (0.69)	–0.36 (0.80)	0.910; 0.437	0.06; 0.47
	**Letters-numbers (WAIS-III)**	–0.08 (0.65)	0.01 (0.91)	0.21 (0.38)	0.372; 0.631	–0.56; –0.29
**Information processing speed**						
	**Total time (TMT-A)**	0.64 (1.14)	0.90 (1.56)	0.61 (0.72)	0.968; 0.695	0.03; 0.24
	**Written score (SDMT)**	–0.06 (0.44)	0.25 (0.24)	0.28 (0.56)	0.296; 0.896	–0.67; –0.07
**Verbal memory**						
	**Long-term free recall (CVLT-II)**	0.40 (0.89)	0.33 (0.60)	–0.08 (1.06)	0.443; 0.425	0.49; 0.48
**Learning**						
	**Total A list (CVLT-II)**	0.60 (0.40)	0.61 (0.88)	0.26 (1.33)	0.607; 0.604	0.33; 0.31
**Executive functioning**						
	**Total time (TMT-B)**	0.60 (0.89)	–0.30 (1.80)	0.33 (1.52)	0.739; 0.526	0.21; –0,38
	**Interference score (Stroop test)**	0.72 (0.52)	0.38 (0.72)	–0.10 (0.84)	0.101; 0.331	1.15; 0.61
	**Total moves (TOL)**	0.70 (0.73)	1.05 (0.81)	–0.61 (1.28)	0.073; 0.022	1.22; 1.55
	**Percentage errors (WCST)**	0.90 (1.11)	0.35 (0.69)	–0.02 (1.28)	0.260; 0.556	0.76; 0.36
**Verbal fluency**						
	**Total score (COWAT)**	0.48 (0.55)	0.23 (0.88)	0.15 (0.42)	0.290; 0.839	0.68; 0.11
	**Total score (Animals test)**	0.06 (0.90)	–0.36 (0.77)	–0.05 (0.62)	0.817; 0.455	0.14; –0.44
**Motor function**						
	**Dominant hand time (GPT)**	0.38 (0.93)	–0.08 (0.71)	–0.21 (0.93)	0.778; 0.545	0.63; 0.15
	**Non-dominant hand time (GPT)**	0.56 (0.92)	–0.16 (0.39)	–0.11 (0.71)	0.202; 0.883	0.82; –0.09
**Global**						
	**NPZ-7**	0.35 (0.14)	0.25 (0.40)	0.20 (0.44)	0.484; 0.849	0.38; 0.12

Values are expressed as mean (standard deviation) except when indicated otherwise.

Higher change scores represent improvement.

Abbreviations: COWAT, Controlled Oral Word Association Test; CVLT-II, California Verbal Learning Test—Version II; GDS, global deficit score; GPT, Grooved Pegboard Test; SDMT, Symbol Digit Modalities Test; TMT-A, Trail Making Test—Part A; TMT-B, Trail Making Test—Part B; WAIS-III, Wechsler Adult Intelligence Scale—Version III; WCST, Wisconsin Card Sorting Test; TOL, Tower of London.

^a^ Comparison between rivastigmine group and control group.

^b^ Comparison between lithium group and control group.

**Table 3:** Change in daily living, quality of life, and emotional variables.

		Rivastigmine (n = 5)	Lithium (n = 6)	Control (n = 6)	*p* Value ^*a*^^;^ ^*b*^	*d* Size ^*a*^^;^ ^*b*^
**Daily living**						
	**Finances**	0.20 (0.83)	0.16 (0.40)	0 (0)	0.568; 0.340	0.36; 0.57
	**Doing the shopping**	0 (0)	0.33 (0.81)	0 (0.63)	1; 0.447	0; 0.45
	**Cooking**	–0.40 (0.54)	0.33 (0.81)	0.33 (0.51)	0.048; 1	–1.39; 0
	**Social activities**	0 (0.70)	0 (0.89)	–0.50 (0.54)	0.218; 0.270	0.81; 0.68
	**Reading/watching TV**	0 (0.70)	0 (0.63)	0.16 (0.40)	0.635; 0.599	–0.29; –0.30
	**Driving**	–0.20 (0.44)	0.16 (0.40)	0 (0)	0.296; 0.340	–0.68; 0.57
	**Using the telephone**	0 (0)	0.16 (0.75)	0.16 (0.75)	0.635; 1	–0.29; 0
	**Home repairs**	0.20 (0.44)	–0.50 (0.83)	0 (0)	0.296; 0.173	0.68; –0.85
	**Going shopping**	0.20 (0.44)	0.33 (0.51)	0 (0.63)	0.568; 0.340	0.36; 0.58
	**Washing clothes**	–0.20 (0.44)	0.16 (0.40)	0.16 (0.40)	0.188; 1	–0.86; 0
	**Medication management**	0 (0)	0 (0)	0 (0.63)	1; 1	0; 0
	**Work**	–0.20 (0.83)	0 (0.63)	0.50 (0.83)	0.200; 0.270	–0.84; –0.68
	**Total impaired areas**	–0.60 (3.71)	1 (4.64)	0.66 (3.72)	0.587; 0.893	–0.34; 0.08
**Quality of life**						
	**Physical dimension**	–0.20 (0.44)	–0.50 (0.83)	0.16 (0.75)	0.365; 0.177	–0.57; –0.83
	**Mental dimension**	0 (0.70)	0.16 (0.98)	0 (0.63)	1; 0.734	0; 0.19
	**Social dimension**	0 (0.70)	0 (1.26)	–0.16 (0.75)	0.715; 0.787	0.22; 0.15
	**Global quality of life**	–0.20 (0.44)	0 (1.26)	0 (0)	0.296; 1	–0.68; 0
**Emotional status**						
	**Depression symptoms**	–1.20 (4.14)	–0.20 (0.83)	–1.20 (3.83)	1; 0.584	0; 0.36
	**Anxiety symptoms**	–1.60 (2.88)	0.60 (5.31)	1.20 (5.54)	0.345; 0.865	–0.61; –0.11

Values are expressed as mean (standard deviation) except when indicated otherwise.

For daily functioning, lower change scores represent improvement.

For quality of life, higher change scores represent improvement.

For emotional status, lower change scores represent improvement.

^a^ Comparison between rivastigmine group and control group.

^b^ Comparison between lithium group and control group.

### Safety outcomes

A total of 12 (41%) participants dropped out of the study. At week 12, 3 (30%) had discontinued in the RG, 2 (18%) in the LG, and 2 (25%) in the CG. The reasons for discontinuation in the RG were low-grade gastrointestinal effects and low-grade fatigue considered related to the study medication in one patient, epistaxis that was considered unrelated to the study medication in another patient, and personal reasons in the third patient. In the LG, one patient discontinued owing to decreased levels of the hormone T4, which indicated low-grade hypothyroidism (0.74 [normal range, 0.82–1.77]), and another developed acute hepatitis B infection, which was considered unrelated to lithium. In the CG, one patient was excluded because of uncompleted assessments, and another developed emotional disturbances. From week 12 to week 48, 2 (20%) patients dropped out in the RG, and 3 (27%) in the LG. In the RG, one withdrew because of patch-related skin irritation, and another developed depression linked to personal problems. In the LG, one patient developed hypothyroidism, another tremors and rash, and the third headache and asthenia. In all 3 cases, the symptoms were considered adverse events associated with lithium. In total, therefore, 2 (20%) participants dropped out because of adverse events in the RG, and 4 (36%) in the LG. Blood tests revealed no significant changes in complete blood count, biochemistry, or immunology, and no other clinical abnormalities were recorded during follow-up.

## Discussion

The results from this small randomized trial show that transdermal rivastigmine was generally well tolerated by HIV-infected patients on stable cART, although no significant improvements in cognitive functioning or other relevant functional parameters were recorded. The study sample comprised mostly highly educated middle-aged MSM who were virologically suppressed, with no potential confounding comorbidities for cognitive impairment.

Trials testing adjuvant therapies for HIV-related neurocognitive complications have been carried out for years. Since the advent of cART, 12 compounds in a total of 18 trials have been investigated.^7^ The therapies included mainly lithium, memantine, minocycline, selegiline, and valproic acid. The studies found no relevant benefits regarding cognitive functioning, except for mild improvements with lithium. Most of the studies prioritized safety and tolerability over efficacy. The studies also revealed discrepancies with regard to the populations studied and the definition of cognitive change. We designed a study to investigate clear efficacy-based clinical endpoints that examined a wide range of cognitive measures and domains. Functional parameters, specifically assessment of daily living, quality of life, and emotional status, have received little attention in previous trials in the field. We analyzed both functional parameters and satisfaction outcomes. Nonetheless, our findings did not reveal relevant differences in any of these dimensions, although a slight trend towards improvement was observed in the rivastigmine arm. The efficacy of rivastigmine has been demonstrated in Alzheimer's and Parkinson's diseases, neurodegenerative processes in which dementia, rather than mild cognitive impairment, is more common [39,40]. Our study sample, while mostly comprising people with mild neurocognitive disorder (MND), did not incorporate cases of HIV-associated dementia; therefore, this cholinesterase may not have been sufficiently potent to achieve an effect on subtle cognitive deficits.

Simioni et al are the only other authors to have previously investigated the effects of rivastigmine in patients with HIV-associated cognitive impairment [14]. After a 20-week study, they concluded that rivastigmine enhanced neurocognitive performance, although this was observed exclusively in 1 measure of information processing speed, out of a total of 12 cognitive measures covering 4 cognitive domains. Our study period was 48 weeks, with an assessment at week 12, which was to some extent comparable to their 20-week assessment. Interestingly, we found information processing speed to be the domain that showed a clearer trend towards improvement. In addition, we detected a significant difference in a measure of attention/working memory (*WAIS-III digit span backward score*), a domain that is known to overlap with information processing speed depending on the tests used [41]. Both effects, however, were lost at week 48, when the only domain with a significantly better score was executive functioning (*TOL total moves measure*), for which Simioni et al found a tendency towards improvement at 20 weeks. Hence, findings from both trials appear to be consistent, despite the absence of statistically significant differences. We also observed an improvement in cognitive outcomes in the 3 study arms, thus indicating that the results could be affected by a practice effect linked to neuropsychological testing. Nevertheless, we incorporated a control group, and our endpoints were predominantly based on the comparison between the medication arms and the control group, rather than a longitudinal approach.

Transdermal rivastigmine has proven to be better tolerated than the oral formulation [8]. Indeed, we found that discontinuation due to side effects was less frequent than Simioni et al (10% vs 23%, respectively), although we assessed this variable 8 weeks earlier. Thirty-six weeks later (at 48 weeks), the rate of discontinuation was similar to that described by Simioni et al, although in their case the assessment was at week 20 (20% vs 23%).

Because most efficacy results in the field were reported from week 10 to 24, we decided to extend our trial to 48 weeks and add an assessment at week 12. We were therefore able to report results that could be compared with previous findings and to better monitor the dynamics of the changes observed. The inclusion of the lithium arm is also a unique characteristic that enabled us to perform 2- and 3-arm comparisons. Furthermore, we used effect size tests to provide unbiased additional information rather than traditional *p* values because of the reduced group sizes. The results supported the trends observed, with some large and very large effects (e.g., in executive functioning). Our trial also benefited from stratification based on the CPE score, which has rarely been monitored in previous studies exploring adjuvant therapies, despite its relevance in the setting of HIV infection.

Our study is subject to a series of limitations. First, the sample was small, with few patients in each group; consequently, results that might have represented more significant changes may have gone undetected. Additionally, despite the randomized controlled design, representation for some baseline variables was uneven. The adjusted analyses subsequently confirmed previous results, but this was not an ideal approach, especially given the original design of the study. Another limitation was the non-blinded character of the trial. Although this approach was intrinsic to the type of investigation—rivastigmine was administered with a transdermal patch and the lithium dosage was adjusted according to drug blood levels—it decreased the methodological power of the design. The trial was also limited by the absence of an extra study assessment between the 12- and the 48-week visits. This could have provided valuable data on the dynamics of study outcomes during follow-up. Furthermore, study efficacy outcomes were established based exclusively on neurocognitive functioning, and neuroimaging parameters or potentially representative CNS biomarkers were not taken into account. Both types of markers are essential when studying therapeutic strategies for HAND.

In summary, the results of this randomized controlled trial suggest that transdermal rivastigmine does not provide a significant cognitive benefit for HIV-infected persons on cART after a 48-week follow-up, despite the tolerability profile was better than that of the oral formulation. Given the fact that ours was a pilot study, the small sample size, and, particularly, the dropout rate, a larger controlled trial should be conducted to contrast our results. In order to ensure appropriate testing of the actual effects of rivastigmine in people with HAND, new trials should include additional markers of CNS functioning and, probably, individuals with more marked cognitive impairment.

## Supporting information

S1 Table CONSORT checklist. (PDF)

S1 Text Original version of the study protocol approved by the ethics committee. (PDF)

S2 Text English adapted version of the study protocol. (PDF)
