# Supplementary material for: Transdermal rivastigmine for HIV-associated cognitive impairment: A randomized pilot study
Source: PLoS One. 2017 Aug 30;12(8):e0182547. doi: 10.1371/journal.pone.0182547 (PMC5576750; doi:10.1371/journal.pone.0182547)
Supplement: S1 Text — (PDF) [file pone.0182547.s002.pdf]

www.flsida.org

Exploratory Controlled Prospective Randomized Trial to Compare the Efficacy and Safety of Two Different Pharmacology Strategies for Neurocognitive Impairment in HIV Infection. TRIANT-TE Study

Code: TRIANT-TE

Version 1, 14th October 2010

EudraCT: 2010-024510-57

Sponsor:

Fundació Lluita contra la SIDA
Hospital Universitari Germans Trias i Pujol
Carretera de Canyet s/n
08916 Badalona (Barcelona)

Principal Investigator: Jose A. Muñoz-Moreno, MS

Co-Investigators:
Anna Prats, MS
Núria Pérez-Álvarez, MS
Carmina R. Fumaz, MS, PhD
Maria J. Ferrer, MS
José Moltó, MD, PhD
Eugènia Negredo, MD, PhD
Bonaventura Clotet, MD, PhD
Crisanto Díez, MD, PhD
Àngels Andreu, PharmD, PhD
Pilar Giner, PharmD, PhD
Maite Garolera, MS, PhD

The information contained in this document is confidential and must not be revealed to third persons without prior authorization as contemplated by Law.

SIGNATURES

The coordinating investigator and the sponsor of the study:
Exploratory Controlled Prospective Randomized Trial to Compare the Efficacy and Safety of Two Different Pharmacology Strategies on Neurocognitive Impairment in HIV Infection. The TRIANT-TE Study

Declare that this study will be conducted in compliance with the protocol, Good Clinical Practices (GCP) and the applicable regulatory requirements.

Modifications to this protocol must be submitted prior agreement of the principal investigator and sponsor.

Principal Investigator: Jose A. Muñoz-Moreno, MS

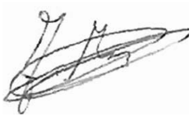

Signature and Date: 5th/January/2011

Sponsor: Bonaventura Clotet, PhD, MD
Fundació Lluita contra la SIDA

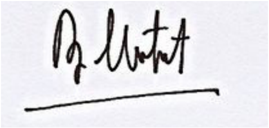

Signature and Date: 5th/January/2011

ANNEX 3, OP.02.01.FLS-RS.02. Pre-estudi 16/06/2015

ANNEX 3, OP.02.01.FLS-RS.02. Pre-estudi 16/06/2015

1 GENERAL INFORMATION

1.1 TITLE

Exploratory Controlled Prospective Randomized Trial to Compare the Efficacy and Safety of Two Different Pharmacology Strategies on Neurocognitive Impairment in HIV Infection. The TRIANT-TE Study

1.2 CODE

TRIANT-TE

1.3 PROTOCOL VERSION AND DATE

Version 6, 17th April 2012. Any modification of the protocol must also bear the amendment number and date.

1.4 SPONSOR

Lluita contra la SIDA Foundation
Germans Trias i Pujol Hospital
Carretera de Canyet s/n
08916 Badalona (Barcelona)
T. 93 465 78 97
F. 93 465 76 02

Person authorized by the sponsor to sign the protocol and amendments:
Bonaventura Clotet Sala, president of Lluita contra la SIDA Foundation

1.5 MONITOR

Jéssica Toro / Silvia Gel
FLS-Research Support
jtoro@fls-rs.com / sgel@fls-rs.com
Germans Trias i Pujol Hospital
Ctra. de Canyet, s/n
08916 Badalona (Barcelona)
T. 93 497 84 14
F. 93 465 76 02

1.6 PRINCIPAL INVESTIGATOR

Jose A. Muñoz-Moreno, MS
HIV Unit, Lluita contra la SIDA Foundation
Hospital Universitari Germans Trias i Pujol, Badalona (Barcelona)
jmunoz@flsida.org

1.7 SITE AND INVESTIGATORS

Lluita contra la SIDA Foundation<br>Ctra. Canyet, s/n<br>08916 Badalona, Barcelona<br>Phone: 93 497 88 87<br>Fax: 93 465 76 02	Anna Prats, MS<br>HIV Unit<br><br>Núria Pérez-Álvarez, MS<br>HIV Unit
Hospital U. Germans Trias i Pujol<br>Ctra. Canyet, s/n<br>08916 Badalona, Barcelona<br>Phone: 93 497 88 87<br>Fax: 93 465 76 02	Carmina R. Fumaz, MS, PhD<br>HIV Unit<br><br>Maria J. Ferrer, MS<br>HIV Unit<br><br>José Moltó, MD, PhD<br>HIV Unit<br><br>Eugènia Negredo, MD, PhD<br>HIV Unit<br><br>Bonaventura Clotet, MD, PhD<br>HIV Unit<br><br>Crisanto Díez, MD, PhD<br>Psychiatry Service<br><br>Àngels Andreu, PharmD, PhD<br>Pharmacy service<br><br>Pilar Giner, PharmD, PhD<br>Pharmacy service
Consorci Sanitari Hospital de Terrassa<br>Ctra. Torrebonica s/n.<br>08227 Terrassa - Barcelona<br>Tel. 93 731 00 07	Maite Garolera, MS, PhD<br>Psychiatry and clinical psychology service.

1.8 TECHNICAL SERVICES INVOLVED

Blood tests and lithium levels will be performed in the laboratories of Hospital Germans Trias i Pujol.

ANNEX 3, OP.02.01.FLS-RS.02. Pre-estudi 16/06/2015

TABLE OF CONTENTS

1 GENERAL INFORMATION 3

TABLE OF CONTENTS 5

2 DESCRIPTION OF THE STUDY 6

3 TYPE OF STUDY 6

4 STUDY ENDPOINTS 6

5 CALENDAR 7

6 STUDY ARMS 7

7 BACKGROUND INFORMATION 8

8 STUDY CRITERIA 8

9 DIRECT ACCESS TO SOURCE DATA/DOCUMENTS 10

10 QUALITY CONTROL AND QUALITY ASSURANCE 10

11 ETHICS 11

12 DATA HANDLING AND RECORD KEEPING 12

13 PUBLICATION POLICY 12

APPENDIX I: CASE REPORT FORM (CRF) 13

APPENDIX II: INVESTIGATOR'S BROCHURE 14

APPENDIX III: PATIENT INFORMATION AND WRITTEN INFORMED CONSENT 15

APPENDIX IV: INSURANCE 16

APPENDIX V: SAE NOTIFICATION 17

ANNEX 3, OP.02.01.FLS-RS.02. Pre-estudi 16/06/2015

2 DESCRIPTION OF THE STUDY

The current project proposes the comparison of two pharmacologic strategies as adjunctive treatments for the improvement of HIV-associated neurocognitive disruption, additionally to use of HAART. The investigators propose the use of the compound that has shown greatest benefits in this context to date, the lithium, versus the use of a well-tolerated and promising drug in other pathologies with neurocognitive affectation, such as Alzheimer or Parkinson diseases, which is the rivastigmine. In those other diseases, this second compound has recently offered a good tolerability, but also benefits on attention, memory and other neurocognitive areas. Both study groups, patients on therapy with lithium and patients on therapy with rivastigmine, will be compared to a control group, which will not initiate any other treatment (therefore only continuing antiretroviral therapy). The investigators are aware that this proposal will offer new relevant data for the study of neurocognitive improvement in HIV infection, as well will allow a better knowledge of clinical management of HIV-infected patients with CNS disease, an aspect that is a common clinical concern today.

3 TYPE OF STUDY

Condition	Intervention	Phase
Neurocognitive Disturbance HIV Infection	Drug: Lithium Drug: Rivastigmine	Phase 4

Study Type: Interventional

Study Design: Allocation: Randomized
Intervention Model: Parallel Assignment
Masking: Open Label
Primary Purpose: Treatment

Official Title: Exploratory Controlled Prospective Randomized Trial to Compare the Efficacy and Safety of Two Different Pharmacology Strategies on Neurocognitive Impairment in HIV Infection.

The TRIANT-TE Study (TRI-ANTiretroviral and Adjuvant ThErapies for HIV-associated cognitive impairment)

4 STUDY ENDPOINTS

- Neurocognitive global composite score: NPZ7 score

  [Time Frame: From screening to month 12]

- Percentage of persons with neurocognitive impairment: 7 cognitive domains (Frascati criteria)

ANNEX 3, OP.02.01.FLS-RS.02. Pre-estudi 16/06/2015

[Time Frame: From screening to month 12]

- Percentage of persons with cognitive complaints (Self-reported)

  [Time Frame: From screening to month 12]

- Functional variables: 12 scales covering functioning in activities of daily living (IADL questionnaire)

  [Time Frame: From screening to month 12]

- Quality of life variables: 4 dimensions assessing quality of life parameters (MOS-HIV questionnaire)

  [Time Frame: From screening to month 12]

- Emotional variables: depression and anxiety symptoms (HADS questionnaire)

  [Time Frame: From screening to month 12]

- Adverse events associated with the initiation of therapy and toxicity parameters

  [Time Frame: Week 2, month 1, 3, 6, 9, 12]

ANNEX 3, OP.02.01.FLS-RS.02. Pre-estudi 16/06/2015

5 CALENDAR

Start Date: April 2011

Completion Date: March 2013

Final Report Date: March 2014

6 STUDY ARMS

Arms	Assigned Interventions
Experimental: Lithium<br>Lithium group: Patients who will initiate therapy with lithium, in tablets, beginning a 2-daily 400 mg dose, and changing further adjusting the dose according to drug levels in serum.	Drug: Lithium<br>lithium, in tablets, beginning a 2-daily 400 mg dose, and changing further adjusting the dose according to drug levels in serum.
Active Comparator: Rivastigmine<br>rivastigmine, in transdermal patch administration, beginning a once-daily 4.6 mg dose, and changing further	Drug: Rivastigmine<br>rivastigmine, in transdermal patch administration, beginning a once-daily 4.6 mg dose, and changing

increasing the dose up to once-daily 9.5 mg.	further increasing the dose up to once-daily 9.5 mg.
No Intervention: Control group<br>Patients who will not initiate treatment	

7 BACKGROUND INFORMATION

Adjunctive treatments based on neurocognitive improvement for HIV-infected patients with CNS disruption have consisted essentially of neurostimulant or neuroprotective treatments. Reports published to date have involved valproic acid, peptide T, CPI-1189, selegiline, memantine, minocycline and lithium. Regarding valproic acid, two trials have confirmed lack of benefit using this compound on HIV-associated neurocognitive decline. In case of peptide T, CPI-1189, selegiline, memantine and minocycline, although their potential mechanisms on brain follow different pathways, trend towards improvement on neurocognitive functioning has been observed. Nonetheless, results on those trials are particularly based on a short term and, moreover, mild connections with benefits on neurocognitive and functional measures have been established. The lithium has been the compound showing clearest benefits on this regard. Two reports have consistently demonstrated benefits on neurocognitive performance using this neuroprotective agent, both in patients with HIV and showing impairment previously. However, lithium is well known to be a drug not easily incorporated in routine practice, at least further than in a psychiatry context. In addition, adverse events related to their use are relatively frequent, and therefore clinical follow-up must be especially controlled. Besides, lithium concentrations are also a concerning aspect considering its use, and drug plasma levels are recommended to be performed throughout the therapy application.

For all these reasons, the current project proposes the comparison of two pharmacologic strategies as adjunctive treatments for the improvement of HIV-associated neurocognitive disruption, additionally to use of HAART. The investigators propose the use of the compound that has shown greatest benefits in this context to date, the lithium, versus the use of a well-tolerated and promising drug in other pathologies with neurocognitive affectation, such as Alzheimer or Parkinson diseases, which is the rivastigmine. In those other diseases, this second compound has recently offered a good tolerability, but also benefits on attention, memory and other neurocognitive areas. Furthermore, in the case of this project, rivastigmine is suggested to be used through a transdermal system patch, a fact that can provide suitability and comfortability with regard to the selected administration method. Both study groups, patients on therapy with lithium and patients on therapy with rivastigmine, will be compared to a control group, which will not initiate any other treatment (therefore only continuing antiretroviral therapy). The investigators are aware that this proposal will offer new relevant data for the study of neurocognitive improvement in HIV infection, as well will allow a better knowledge of clinical management of HIV-infected patients with CNS disease, an aspect that is a common clinical concern today.

8 STUDY CRITERIA

Inclusion Criteria:

- Age ranged from 20 to 75 years old

ANNEX 3, OP.02.01.FLS-RS.02. Pre-estudi 16/06/2015

- Correct understanding of study objectives
- Written consent signed
- HIV infection confirmed by Western Blot or two ELISA tests
- Existence of an HIV-associated neurocognitive disorder according to the diagnosis classification offered by Antinori and cols (Neurology, 2007)
- Being on antiretroviral treatment.
- Spanish/Catalan speaker.

Exclusion Criteria:

- To be on a treatment that may interact pharmacologically with any of the new drugs used in study arms.
- Breastfeeding, pregnancy or fertile women willing to be pregnant.
- Renal failure or severe cardiovascular disease.
- Weakness, dehydration or severe sodium depletion.
- Sick sinus syndrome or cardiac conduction disturbances (sinoatrial block or atrioventricular block).
- Active duodenal or gastric ulcer.
- Urinary obstruction.
- Epilepsy.
- Chronic obstructive pulmonary disease (COPD).

ANNEX 3, OP.02.01.FLS-RS.02. Pre-estudi 16/06/2015

ANNEX 3, OP.02.01.FLS-RS.02. Pre-estudi 16/06/2015

9 DIRECT ACCESS TO SOURCE DATA/DOCUMENTS

Investigators and institutions will allow the monitoring, and audits by the Health Authorities or the Sponsor giving direct access to data and original source documents.

Access to personal patient information will be restricted to the Study physician / staff. To allow monitorings, audits and inspections, access to data to Health Authorities (Spanish Agency for Medicines and Health Products), the Ethics Committee and personnel authorized by the Sponsor, is guaranteed while maintaining the confidentiality thereof according to current legislation.

10 QUALITY CONTROL AND QUALITY ASSURANCE

10.1 STUDY MONITORING

In accordance with applicable regulations and Good Clinical Practice (GCP), the monitor will visit or contact the center on a regular basis. The duration, nature and frequency of visits / contacts depend on the monitoring plan.

During these contacts, the monitor shall:

- monitor and evaluate the progress of the study;
- examining the data collected;
- carry out a verification of the source documents;
- identify any problems and find solutions;

The goal of the monitoring activity is to verify that:

- the rights and welfare of subjects are respected;
- survey data are accurate, complete and verifiable with the help of original documents;
- the study is performed according to the protocol and any amendment adopted, GCPs and regulations.

The investigator must agree to:

- grant to monitor direct access to all relevant documentation;
- devote part of his/her time and staff time to the monitor in order to discuss the results of the monitoring, as well as any other possible aspect.

The monitor should also contact the center before starting the study with the aim to discuss with staff the Protocol and procedures for data collection.

10.2 AUDITS AND INSPECTIONS

Sponsor can carry out an audit of quality control at its sole discretion. In this case, the investigator should agree to grant the auditor direct access to all relevant documentation and devote part of his/her time and staff time to the auditor in order to discuss the results of the monitoring, as well as any other possible aspect.

ANNEX 3, OP.02.01.FLS-RS.02. Pre-estudi 16/06/2015

Moreover, regulatory authorities may also inspect the study. In this case, the investigator should agree to give the inspector direct access to all relevant documentation and devote part of his/her time and staff time to the inspector in order to discuss the results of the supervision, as well as any other possible aspect.

10.3 CASE REPORT FORM

Data collection will be done through an electronic CRF with a system of access by username and password. The application has track changes (recording the user that has performed).

Accurate and reliable data collection is ensured by checking and cross checking the CRF front site records conducted by the study monitor (verification of source documents). The data collected will be added to a computer database which will be reviewed for possible inconsistencies to be resolved by the research team of the study in each site.

The content of the CRF is attached in Appendix I.

11 ETHICS

11.1 GENERAL CONSIDERATIONS

The clinical trial will be conducted according to the principles of the Declaration of Helsinki.
This study will be conducted according to Spanish regulations and the required documentation prior to the start will be:

- Protocol acceptance by the sponsor and the coordinating investigator
- Protocol approval by the Ethics Committee.
- Protocol authorization from the Spanish Drug Agency (Ministry of Health)

All subjects will be guaranteed continued medical and nursing supervision throughout the duration of the study.

This study will conform to the standards of "Good Clinical Practice".

11.2 PATIENT INFORMATION SHEET AND INFORMED CONSENT

Informed consent will be obtained before including the patient in the trial (Appendix III). The investigator is to inform the patient of the nature, duration and purpose of the study, as well as of all the obstacles and inconveniences which – within reason – may be expected from it. Furthermore, the patient is to receive information in writing. The participating patients must be legally competent to give informed consent, with the possibility of taking decisions at his/her own free will. The patient has the right to leave the study at any time.

ANNEX 3, OP.02.01.FLS-RS.02. Pre-estudi 16/06/2015

12 DATA HANDLING AND RECORD KEEPING

12.1 DATA HANDLING

The processing of the data to be compiled by the study sponsor during the trial will be subject to current legislation as regards data protection (LOPD, Ley Orgánica 15/1999, de 13 de diciembre de protección de datos de carácter personal). The patient will be identified in the records by the corresponding code number only. The patient is to be guaranteed anonymity, and is to be informed that all communication will take place between him/her and the investigator – not the sponsor of the trial.

Data transmitted to third countries and other countries will in no case contain personal data. In the event that such transfer occurs, it will be for the same purposes of the study described and ensuring confidentiality at least to the level of protection of the law in Spain.

12.2 RECORD KEEPING

12.2.1 Investigator file and document retention

The investigator must keep the investigator file with the proper and accurate records to enable the study to be fully documented and data subsequently verified.

The Investigator's study file will contain the protocol and its amendments, CRFs, questionnaires' forms, EC approval and authorization from the health authorities, samples of the patient information sheet and informed consent, staff curriculum, signatures' delegation log and listing of subjects, as well as other appropriate documents and correspondence.

Clinical source documents from subjects (usually predefined by the project to record key efficacy and safety parameters or documents that are not in the clinical record of the hospital) will be filed indicating the number of patient without personal data.

The investigator should retain these documents at least twenty five years, according to Royal Decree 1090/2015, provided that the sponsor does not express another period.

12.2.2 Source documents and basic data

Patient participation in the study will be included on medical records, including assigned code number and identification of the different study visits that will take place throughout the study. At the end of the study, a copy of the CRF will be placed on the site.

13 PUBLICATION POLICY

The publication of the trial results shall meet the requirements set out in Article 38 of Royal Decree 223/2004.

APPENDIX I: CASE REPORT FORM (CRF)

ANNEX 3, OP.02.01.FLS-RS.02. Pre-estudi 16/06/2015

APPENDIX II: INVESTIGATOR'S BROCHURE

ANNEX 3, OP.02.01.FLS-RS.02. Pre-estudi 16/06/2015

APPENDIX III: PATIENT INFORMATION AND WRITTEN INFORMED CONSENT

ANNEX 3, OP.02.01.FLS-RS.02. Pre-estudi 16/06/2015

APPENDIX IV: INSURANCE

ANNEX 3, OP.02.01.FLS-RS.02. Pre-estudi 16/06/2015

APPENDIX V: SAE NOTIFICATION

ANNEX 3, OP.02.01.FLS-RS.02. Pre-estudi 16/06/2015
