# Supplementary material for: Transdermal rivastigmine for HIV-associated cognitive impairment: A randomized pilot study
Source: PLoS One. 2017 Aug 30;12(8):e0182547. doi: 10.1371/journal.pone.0182547 (PMC5576750; doi:10.1371/journal.pone.0182547)
Supplement: S2 Text — (PDF) [file pone.0182547.s003.pdf]

Estudio Randomizado, Prospectivo, Controlado, para Comparar la Eficacia y Seguridad de Dos Estrategias Farmacológicas Diferentes sobre la Alteración Neurocognitiva en la Infección por VIH. Estudio TRIANT-TE.

Código: TRIANT-TE

Versión 1, de 14 de Octubre del 2010

Nº EudraCT: 2010-024510-57

Promotor:

Fundació Lluita contra la SIDA
Hospital Universitari Germans Trias i Pujol
Carretera de Canyet s/n
08916 Badalona (Barcelona)

Investigador Principal: Jose A. Muñoz-Moreno, MS

Co-Investigadores:

Anna Prats, MS
Núria Pérez-Álvarez, MS
Carmina R. Fumaz, MS, PhD
Maria J. Ferrer, MS
Eugènia Negredo, MD, PhD
José Moltó, MD, PhD
Bonaventura Clotet, MD, PhD
Crisanto Díez, MD, PhD
Àngels Andreu, PhD
Pilar Giner, PhD
Maite Garolera, MS, PhD

La información contenida en este documento es confidencial y no debe ser revelada a terceros salvo para obtener las autorizaciones previstas por la Ley.

FIRMA DEL INVESTIGADOR PRINCIPAL Y COLABORADORES

Los investigadores responsables del ensayo clínico:
Estudio Randomizado, Prospectivo, Controlado, para Comparar la Eficacia y Seguridad de Dos Estrategias Farmacológicas Diferentes sobre la Alteración Neurocognitiva en la Infección por VIH. Estudio TRIANT-TE.

Declaran que el ensayo clínico será realizado en cumplimiento de las Buenas Prácticas Clínicas, siguiendo el este protocolo.

Las modificaciones a este protocolo deberán presentarse previa conformidad de los investigadores coordinador/principal y del promotor.

Investigador principal / coordinador: Jose A. Muñoz-Moreno, MS

Firma y fecha 05-ENE-2011

Promotor: Bonaventura Clotet, MD, PhD
Fundació Lluita contra la SIDA

Firma y fecha 05-ENE-2011

1.- RESUMEN

1.0. TIPO DE SOLICITUD

Ensayo clínico aleatorizado y prospectivo con especialidades farmacéuticas en condiciones de uso distintas a las autorizadas.

1.1. IDENTIFICACIÓN DEL PROMOTOR

Fundació de Lluita Contra la SIDA

1.2. TÍTULO DEL ESTUDIO

Estudio Randomizado, Prospectivo, Controlado, para Comparar la Eficacia y Seguridad de Dos Estrategias Farmacológicas Diferentes sobre la Alteración Neurocognitiva en la Infección por VIH. Estudio TRIANT-TE.

1.3. CÓDIGO DEL PROTOCOLO

TRIANT-TE

1.4. INVESTIGADOR PRINCIPAL

Jose A. Muñoz-Moreno, MS
Unitat de VIH
Hospital Universitari Germans Trias i Pujol, Badalona (Barcelona)

1.5. CENTROS PROVISIONALES DE REALIZACIÓN DEL ESTUDIO

Hospital Universitari Germans Trias i Pujol, Badalona (Barcelona)

1.6. COMITÉS ÉTICOS IMPLICADOS

CEIC Hospital Universitari Germans Trias i Pujol

1.7. MONITOR

Jéssica Toro / Silvia Gel
Fundació de Lluita Contra la SIDA
Tel. 93 497 84 14
Fax. 93 465 76 02

1.8. FÁRMACO EXPERIMENTAL

Litio: formulación de comprimidos, inicialmente con una dosis de 400 mg. 2 veces al día y posteriormente ajustando la dosis según controles séricos de niveles de fármaco.

Rivastigmina: formulación de parche transdérmico, iniciando con una dosis de 4,6 mg. una vez al día y posteriormente aumentando la dosis a 9,5 mg. una vez al día.

1.9. FASE DEL ENSAYO CLÍNICO

Fase IV

1.10. OBJETIVO DEL ESTUDIO

Evaluar la eficacia y seguridad del tratamiento con litio y del tratamiento con rivastigmina sobre el funcionamiento neurocognitivo en pacientes infectados por el VIH.

1.11. DISEÑO DEL ESTUDIO

Estudio unicéntrico, aleatorizado, prospectivo, controlado, abierto, con tratamiento activo, paralelo de 12 meses de seguimiento, que comparará la eficacia y seguridad de dos estrategias farmacológicas centradas en la mejora del funcionamiento del SNC en personas infectadas por el VIH y con alteración neurocognitiva.

1.12. PATOLOGÍA DEL ESTUDIO

Alteración neurocognitiva en la infección por VIH.

1.13. VARIABLE PRINCIPAL DE VALORACIÓN

Serán 2 las variables principales de valoración: el porcentaje de pacientes con alteración neurocognitiva y el cambio en un índice global de alteración.

1.14. TIPO DE POBLACIÓN Y NÚMERO DE PACIENTES

Los sujetos que participarán en esta investigación serán pacientes visitados en la Unidad de VIH del Hospital Universitari Germans Trias i Pujol, a los cuales se les informará sobre los procedimientos y objetivos del estudio, y que voluntariamente aceptarán su participación.

El número de sujetos experimentales propuesto para el estudio es de 15 individuos por rama (un total de 45 participantes). Este tamaño muestral ha sido calculado estadísticamente y posteriormente comprobado en función de la potencia asistencial y de inclusión de participantes en ensayos clínicos de la unidad donde se realizará la investigación. Si se incluye un total de 45 unidades experimentales, distribuidas entre los 3 grupos según el ratio 1:1:1, se obtendrá una potencia del 74,2% para detectar diferencias en el contraste de la hipótesis nula, la cual considera que las proporciones de los 3 grupos serán iguales, contra la hipótesis alternativa, la cual considera no igualdad mediante una prueba Chi cuadrado para 3 muestras independientes, teniendo en cuenta que el nivel de significación es del 5%, y asumiendo que los porcentajes esperados de respuesta en los 3 grupos son, aproximadamente, de 75% en la rama de litio, 75% en la de rivastigmina, y 15% en el grupo control. Teniendo en cuenta este resultado, y en base a la estimación de pacientes visitados anualmente y reclutados en ensayos clínicos, el número resultante de 45 participantes se muestra estadísticamente favorable y clínicamente alcanzable.

1.15. DURACIÓN DEL TRATAMIENTO

La duración prevista del tratamiento será de 12 meses.

1.16. CALENDARIO Y FECHA DE FINALIZACIÓN

Fecha de inicio: Abril 2011
Fecha de fin: Marzo 2013
Fecha de presentación del informe final: Marzo 2014

2.- ÍNDICE

1.- RESUMEN **3**
1.0. TIPO DE SOLICITUD 3
1.1. IDENTIFICACIÓN DEL PROMOTOR 3
1.2. TÍTULO DEL ESTUDIO 3
1.3. CÓDIGO DEL PROTOCOLO 3
1.4. INVESTIGADOR PRINCIPAL 3
1.5. CENTROS PROVISIONALES DE REALIZACIÓN DEL ESTUDIO 3
1.6. COMITÉS ÉTICOS IMPLICADOS 3
1.7. MONITOR 3
1.8. FÁRMACO EXPERIMENTAL 3
1.9. FASE DEL ENSAYO CLÍNICO 4
1.10. OBJETIVO DEL ESTUDIO 4
1.11. DISEÑO DEL ESTUDIO 4
1.12. PATOLOGÍA DEL ESTUDIO 4
1.13. VARIABLE PRINCIPAL DE VALORACIÓN 4
1.14. TIPO DE POBLACIÓN Y NÚMERO DE PACIENTES 4
1.15. DURACIÓN DEL TRATAMIENTO 4
1.16. CALENDARIO Y FECHA DE FINALIZACIÓN 5

2.- ÍNDICE **6**

3.- INFORMACIÓN GENERAL **8**
3.1. IDENTIFICACIÓN DEL ENSAYO 8
3.2. TIPO DE ENSAYO 8
3.3. DESCRIPCIÓN DE LOS PRODUCTOS EN ESTUDIO 8
3.4. DATOS RELATIVOS AL PROMOTOR 8
3.5. DIRECTOR TÉCNICO RESPONSABLE DE LA ELABORACIÓN Y CONTROL DEL PRODUCTO EN INVESTIGACIÓN 8
3.6. IDENTIFICACIÓN DEL MONITOR 8
3.7. DATOS DE LOS CENTROS E INVESTIGADORES PARTICIPANTES EN EL ENSAYO 9
3.8. SERVICIOS TÉCNICOS IMPLICADOS 9
3.9. DURACIÓN PREVISTA DEL ENSAYO 9

4.- JUSTIFICACIÓN Y OBJETIVOS **10**
4.1. JUSTIFICACIÓN 10
4.2. OBJETIVOS 12
4.2.1. Objetivo principal 12
4.2.2. Objetivos secundarios 12

5.- TIPO DE ENSAYO CLÍNICO Y DISEÑO **13**
5.1. FASE DE DESARROLLO DEL ENSAYO CLÍNICO 13
5.2. DISEÑO DEL ENSAYO CLÍNICO. 13
5.3. PROCESO DE ALEATORIZACIÓN Y ESTRATIFICACIÓN 13
5.4. GRUPOS DE TRATAMIENTO Y DOSIS 13
5.5. PLAN DEL ESTUDIO, DEFINICIÓN DE ANÁLISIS INTERMEDIOS 14
5.6. TÉCNICA DE ENMASCARAMIENTO 14
5.7. CUADERNO DE RECOGIDA DE DATOS (CRD) 15

6.- SELECCIÓN DE SUJETOS **16**
6.1. CRITERIOS PARA EL DIAGNÓSTICO DE LA PATOLOGÍA EN ESTUDIO 16
6.2. CRITERIOS DE INCLUSIÓN 16
6.3. CRITERIOS DE EXCLUSIÓN 16
6.4. NÚMERO DE SUJETOS 16
6.5. CRITERIOS DE RETIRADA Y ABANDONO 16
6.5.1. Actuación médica ante la retirada 17
6.6. PÉRDIDAS PREALEATORIZACIÓN 17
6.7. DURACIÓN APROXIMADA DEL PERIODO DE RECLUTAMIENTO 17

7.- DESCRIPCIÓN DEL TRATAMIENTO 18
7.1. TRATAMIENTOS EXPERIMENTAL Y CONTROL........ 18
7.2. SUMINISTRO, ACONDICIONAMIENTO, ETIQUETADO Y ALMACENAMIENTO 18
7.3. DOSIS, INTERVALO, VÍA Y FORMA DE ADMINISTRACIÓN 18
7.4. MODIFICACIÓN DE LA PAUTA DE TRATAMIENTO 19
7.5. TRATAMIENTOS CONCOMITANTES 19
7.6. CUMPLIMIENTO 21
7.7. NORMAS PARA EL MANEJO DE LOS TRATAMIENTOS DE ESTUDIO 21

8.- DESARROLLO DEL ENSAYO Y EVALUACIÓN DE LA RESPUESTA 22
8.1. CRITERIOS PARA LA EVALUACIÓN DE LA RESPUESTA 22
8.1.1. VARIABLE PRINCIPAL A ESTUDIAR 22
8.1.2.VARIABLES SECUNDARIAS A ESTUDIAR........ 22
8.1.3. ENDPOINTS DEL ESTUDIO 23
8.2. PROCEDIMIENTOS DE LOS PARÁMETROS A EVALUAR 24
8.2.1. Historia clínica y variables demográficas........ 24
8.2.2. Análisis de laboratorio 24
8.3. DESARROLLO DEL ENSAYO 25

9.- ACONTECIMIENTOS ADVERSOS 26
9.1. DEFINICIÓN 26
9.2. DESCRIPCIÓN DE LOS CRITERIOS DE IMPUTABILIDAD 26
9.3. PROCEDIMIENTO PARA LA NOTIFICACIÓN DE LOS ACONTECIMIENTOS ADVERSOS........ 27

10.- ASPECTOS ÉTICOS 29
10.1. CONSIDERACIONES GENERALES........ 29
10.1.1. Declaración de Helsinki 29
10.1.2. Comités éticos y autoridad reguladora........ 29
10.2. INFORMACIÓN AL PACIENTE Y CONSENTIMIENTO INFORMADO 29
10.3. CONFIDENCIALIDAD. PROTECCIÓN DEL PACIENTE 29

11.- CONSIDERACIONES PRÁCTICAS 30
11.1. RESPONSABILIDADES DE LOS PARTICIPANTES EN EL ESTUDIO 30
11.2. MODIFICACIONES DEL PROTOCOLO 30
11.3. RECOGIDA Y ARCHIVO DE DATOS 30
11.4. BUENA PRÁCTICA CLÍNICA........ 31
11.5 PUBLICACIÓN 31
11.6. DOCUMENTACIÓN DEL ESTUDIO........ 31

12.- ANÁLISIS ESTADÍSTICO 32
12.1. POBLACIONES PARA EL ANÁLISIS 32
12.2. DESCRIPCIÓN DE LA MUESTRA........ 32
12.3. ANÁLISIS DE EFICACIA 33
12.4. ANÁLISIS DE SEGURIDAD........ 33
12.5. DESCRIPCIÓN DEL TAMAÑO DE LA MUESTRA........ 33

13. REFERENCIAS........ 34

ANEXO I: CUADERNO DE RECOGIDA DE DATOS (CRD) 36

ANEXO II: MANUAL DEL INVESTIGADOR........ 37

ANEXO III: HOJA DE INFORMACIÓN AL PACIENTE Y CONSENTIMIENTO INFORMADO 38

ANEXO IV: SEGURO........ 39

ANEXO V: FORMULARIO DE NOTIFICACIÓN DE SAE 40

3.- INFORMACIÓN GENERAL

3.1. IDENTIFICACIÓN DEL ENSAYO

Código: TRIANT-TE

Título: Estudio Randomizado Prospectivo Controlado para Comparar la Eficacia y Seguridad de Dos Estrategias Farmacológicas Diferentes sobre la Alteración Neurocognitiva en la Infección por VIH. Estudio TRIANT-TE.

3.2. TIPO DE ENSAYO

Ensayo clínico aleatorizado y prospectivo con especialidades farmacéuticas en condiciones de uso distintas a las autorizadas.

3.3. DESCRIPCIÓN DE LOS PRODUCTOS EN ESTUDIO

Productos en estudio:

- Litio: en formulación de comprimidos, inicialmente con una dosis de 400 mg. 2 veces al día y posteriormente ajustando la dosis según controles séricos de niveles de fármaco.
- Rivastigmina: en formulación de parche transdérmico, iniciando con una dosis de 4,6 mg. una vez al día y posteriormente aumentando la dosis a 9,5 mg. una vez al día.

La composición de la formulación de cada principio activo corresponde a su formulación comercial.

3.4. DATOS RELATIVOS AL PROMOTOR

Fundació de Lluita Contra la SIDA
Carretera de Canyet, s/n
08916 – Badalona (Barcelona)
Teléfono: 93 497 88 87
Fax: 93 465 76 02
www.flsida.org

3.5. DIRECTOR TÉCNICO RESPONSABLE DE LA ELABORACIÓN Y CONTROL DEL PRODUCTO EN INVESTIGACIÓN

Los del laboratorio fabricante ya que se usará medicación comercial.

3.6. IDENTIFICACIÓN DEL MONITOR

Jéssica Toro/Silvia Gel
FLS-Research Support
Carretera de Canyet, s/n
08916 – Badalona (Barcelona)
Teléfono: 93 497 84 14, [Móbil monitora]
Fax: 93 465 76 02
jtoro@fls-rs.com

3.7. DATOS DE LOS CENTROS E INVESTIGADORES PARTICIPANTES EN EL ENSAYO

Investigador principal:Jose A. Muñoz-Moreno, MS (jmunoz@flsida.org)

Investigadores colaboradores:

Fundació Lluita contra la SIDA<br>Ctra. Canyet, s/n<br>08916 Badalona, Barcelona<br>Teléfono: + 34 93 497 88 87<br>Fax: + 34 93 465 76 02	Anna Prats, MS<br>Unidad de VIH<br><br>Núria Pérez-Álvarez, MS<br>Unidad de VIH
Hospital U. Germans Trias i Pujol<br>Ctra. Canyet, s/n<br>08916 Badalona, Barcelona<br>Teléfono: + 34 93 497 88 87<br>Fax: + 34 93 465 76 02	Carmina R. Fumaz, PhD<br>Unidad de VIH<br><br>Maria J. Ferrer, MS<br>Unidad de VIH<br><br>Eugènia Negredo, MD, PhD<br>Unidad de VIH<br><br>José Moltó, MD, PhD<br>Unidad de VIH<br><br>Bonaventura Clotet, MD, PhD<br>Unidad de VIH<br><br>Crisanto Díez, MD, PhD<br>Servicio de Psiquiatría<br><br>Àngels Andreu, PhD<br>Servicio de Farmacia<br><br>Pilar Giner, PhD<br>Servicio de Farmacia
Consorci Sanitari Hospital de Terrassa<br>Ctra. Torrebonica s/n.<br>08227 Terrassa - Barcelona<br>Tel. 93 731 00 07	Maite Garolera, MS, PhD<br>Servicio de Psiquiatría y Psicología Clínica

3.8. SERVICIOS TÉCNICOS IMPLICADOS

Para este ensayo se contará con la colaboración de los Laboratorios del Hospital Germans Trias i Pujol para la realización de las analíticas establecidas en el cronograma y la determinación de los niveles de litio para el ajuste de dosis.

3.9. DURACIÓN PREVISTA DEL ENSAYO

La duración máxima del ensayo será de 96 semanas: 48 semanas de inclusión y 48 semanas de seguimiento.

4.- JUSTIFICACIÓN Y OBJETIVOS

4.1. JUSTIFICACIÓN

La alteración del funcionamiento neurocognitivo es una complicación frecuente en la infección por VIH. Diferentes estudios han confirmado que alrededor de la mitad de personas con VIH presentan problemas en su funcionamiento neuropsicológico, implicando el aprendizaje, la memoria de trabajo, la atención, el funcionamiento ejecutivo y la psicomotricidad, como áreas más alteradas en este perfil de afectación (1-5). Además, esto no ha sido relacionado sólo con consecuencias negativas de diferente origen, tales como el empeoramiento del desarrollo de las actividades de la vida diaria o de la calidad de vida (6-8), sino también con factores clínicos, como un mayor fallo virológico (9), o mayores tasas de muerte en aquellas personas con VIH que presentaron previamente cambios neurocognitivos (10).

Diversos factores han provocado esta realidad. Entre ellos, la aparición de nuevos factores de riesgo, como el envejecimiento de las personas infectadas (11,12), recuentos nadir más bajos de células CD4 (13,14), o la coinfección con VHC (15). Sin embargo, relacionado de forma directa con esta elevada prevalencia, destaca también la insuficiente efectividad de las terapias antirretrovirales para mejorar tal situación (16). Concretamente se sabe que el virus persiste en el sistema nervioso central (SNC) a pesar de la eficacia del tratamiento en sangre, y esto ha sido relacionado con una mayor existencia de trastornos neurocognitivos (17), así como con una mayor neuroinflamación asociada al virus, y a otros procesos neuropatogénicos algo más complejos (18-20). Por todos estos motivos, encontrar un tratamiento para los trastornos neurocognitivos asociados al VIH se ha convertido en una necesidad relevante tanto a nivel clínico, como a nivel de investigación.

Diferentes líneas se están desarrollando en el terreno de la búsqueda de estrategias para mejorar el deterioro neurocognitivo por VIH. Por un lado, las estrategias que más comúnmente han mostrado beneficios han sido las propias terapias antirretrovirales (16,21-23). Hoy sabemos que factores como el inicio más temprano de la terapia antirretroviral (24-26), o la no interrupción del tratamiento (27), están asociados ambos con una menor prevalencia de deterioro cognitivo. Además, actualmente se están llevando a cabo diferentes trabajos con el objetivo de encontrar pautas antirretrovirales que penetren mejor la barrera hematoencefálica y, por tanto, que puedan ofrecer más beneficios en la mejora del funcionamiento del SNC (28). Por otro lado, como línea complementaria de actuación, se están estudiando estrategias farmacológicas con origen diferente al de controlar el propio virus. El razonamiento para el uso de estas otras intervenciones ha sido la búsqueda de tratamientos farmacológicos que hayan mostrado beneficios en la alteración neurocognitiva, aunque asociada a otras enfermedades. Algunos ejemplos son la esquizofrenia, la esclerosis múltiple, la enfermedad de Parkinson, o la enfermedad de Alzheimer, donde la afectación neurocognitiva ha sido mejorada gracias a nuevas estrategias farmacológicas. En la infección por VIH, en cambio, existen pocos estudios publicados usando estas otras intervenciones, y con resultados poco contrastados.

En general, los tratamientos adicionales a la terapia antirretroviral que se han centrado en la mejora del funcionamiento neurocognitivo en la infección con VIH, han sido tratamientos neuroestimuladores y/o neuroprotectores. Estos han consistido principalmente en el ácido valproico (29), la selegilina (30), la memantina (31) y el litio (32,33). Más concretamente, los que han mostrado mayores beneficios han sido el ácido valproico y en especial el litio, sobre el cual han sido publicados dos trabajos. Las ventajas de la memantina han sido observadas hasta el momento a corto plazo (existe un estudio en marcha evaluando sus posibles efectos más a largo plazo), y en cuanto a la selegilina, no se han encontrado beneficios significativos relacionados con la mejora neuropsicológica en VIH.

Por otro lado, cabe destacar que estos componentes, incluso los más efectivos, no son fármacos fácilmente aplicables a la rutina clínica. Tanto el ácido valproico como el litio presentan un perfil de efectos secundarios asociados a su uso superior a otros fármacos neuroestimuladores. Ése ha sido el motivo principal por el cual se ha llevado a cabo un estudio con selegilina, en formulación transdermal, aunque sin demostrar efectividad hasta el momento. Además, no sólo la toxicidad es una desventaja en estos fármacos, sino que también lo es la comodidad. Tanto el ácido valproico como el litio requieren controles adicionales en la práctica clínica, tales como pruebas hepáticas en el caso del primero, o niveles farmacológicos en el caso del segundo. Es por todos estos motivos que la necesidad de hallar fármacos tanto seguros, como fáciles de incorporar en la rutina clínica del manejo de los pacientes con VIH, se ha convertido en un aspecto trascendental en este terreno.

Recientemente han sido descubiertos otros fármacos que han mostrado beneficios en el tratamiento de los trastornos neurocognitivos en diversas enfermedades: los inhibidores de la acetilcolinesterasa. Esta familia de fármacos, de los cuales se han comercializado hasta el momento tres compuestos, han mostrado gran eficacia retrasando los efectos del deterioro neuropsicológico, así como un perfil bastante adecuado de tolerabilidad. Además, a pesar de que probablemente el mayor impacto de estos fármacos ha estado vinculado a su uso y beneficios en la enfermedad de Alzheimer (34,35), en la actualidad han sido estudiados en el contexto de otras patologías con perfiles de alteración neurocognitiva, como la esquizofrenia (37), la esclerosis múltiple (38), o la enfermedad de Parkinson (39), y en todas ellas han mostrado adecuada eficacia y seguridad.

Por todas estas razones, el siguiente proyecto plantea la comparación de dos estrategias farmacológicas como tratamientos adicionales y concomitantes al uso del tratamiento antirretroviral en la búsqueda de la mejora del funcionamiento del SNC. Proponemos la comparación de un grupo de estudio en tratamiento con litio, otro grupo en tratamiento con rivastigmina, y ambos serán comparados con un tercer grupo control, que no iniciará ningún tratamiento adicional. Sin duda, somos conscientes de que la propuesta que proponemos ofrecerá nuevos datos en la búsqueda de la mejora neurocognitiva en la infección con VIH, así como permitirá un mejor conocimiento del manejo clínico de estos pacientes, por desgracia sufriendo aún tan frecuentemente este tipo de alteraciones neurológicas.

Hipotesis del estudio

- Tanto el tratamiento con litio como el tratamiento con rivastigmina ayudarán a mejorar el funcionamiento neurocognitivo de pacientes infectados por el VIH que presenten alteración neurocognitiva.

- Ambos tratamientos estarán asociados a una mejora adicional de la realización de actividades de la vida diaria y de la calidad de vida.

- La satisfacción respecto el tratamiento de los pacientes en terapia con rivastigmina será superior a la de los pacientes con litio.

4.2. OBJETIVOS

4.2.1. Objetivo principal

Evaluar la eficacia del tratamiento con litio y del tratamiento con rivastigmina sobre el funcionamiento neurocognitivo en pacientes infectados por el VIH.

4.2.2. Objetivos secundarios

- Valorar la frecuencia de acontecimientos adversos asociados al inicio del tratamiento con litio y del tratamiento con rivastigmina.

- Valorar la satisfacción respecto el tratamiento en ambos grupos de estudio.

- Valorar la mejora funcional en ambos grupos de estudio.

- Valorar la calidad de vida en ambos grupos de estudio.

5.- TIPO DE ENSAYO CLÍNICO Y DISEÑO

5.1. FASE DE DESARROLLO DEL ENSAYO CLÍNICO.

Fase IV

5.2. DISEÑO DEL ENSAYO CLÍNICO.

Estudio unicéntrico, randomizado, prospectivo, controlado, abierto, con tratamiento activo, paralelo de 12 meses de seguimiento, que comparará la eficacia y seguridad de dos estrategias farmacológicas centradas en la mejora del funcionamiento del SNC en personas infectadas por el VIH y con alteración neurocognitiva.

5.3. PROCESO DE ALEATORIZACIÓN Y ESTRATIFICACIÓN

La tabla de aleatorización se realizará mediante una distribución uniforme y asignando un rango de valores a cada uno de los grupos. Concretamente, se llevará a cabo una aleatorización 1:1:1 a 3 ramas de estudio.

A su vez, la randomización será estratificada según el índice estimado de penetrabilidad en el SNC que presente el tratamiento antirretroviral de cada paciente, de acuerdo a la clasificación validada por Letendre y cols (Conference of Retroviruses and Opportunistic Infections, 2010). Siguiendo la recomendación de los propios autores, un índice de <8 se considerará como una pauta poco neuroactiva y ≥8 como altamente neuroactiva. De esta forma la distribución de tratamientos antirretrovirales en función de su índice de penetrabilidad en el SNC estará equilibrada entre los diferentes grupos.

La asignación se realizará telefónicamente y de forma centralizada. De este modo será impredecible para los investigadores antes de la inclusión el grupo al que se asignará el paciente.

5.4. GRUPOS DE TRATAMIENTO Y DOSIS

Habrá tres grupos de tratamiento:

- Grupo litio: Pacientes que iniciarán tratamiento con litio, en formulación de comprimidos, inicialmente con una dosis de 400 mg. 2 veces al día y posteriormente ajustando la dosis según controles séricos de niveles de fármaco.

- Grupo rivastigmina: Pacientes que iniciarán tratamiento con rivastigmina, en formulación de parche transdérmico, iniciando con una dosis de 4,6 mg. una vez al día y posteriormente aumentando la dosis a 9,5 mg. una vez al día.

- Grupo control: Pacientes que no iniciarán tratamiento.

Específicamente, en los pacientes que inicien tratamiento con litio la dosis variará entre ellos, y se ajustará en función de la respuesta del sujeto y de los valores de litemia. Los niveles séricos de litio se determinarán en cada una de las visitas con el objetivo de estabilizar los niveles de fármaco en sangre. Las dosis habituales están entre 1 y 4 comprimidos al día, lo que proporciona unas litemias de 0,7 y 1,2 mEq/l. La litemia mínima eficaz recomendada oscila de 0,5 a 0,8 mEq/l, y la máxima no debe sobrepasar

los 2 mEq/l. En los pacientes que inicien tratamiento con rivastigmina se iniciará tratamiento con una dosis de 4,6 mg. una vez al día y en una semana se aumentará la dosis a 9,5 mg.

5.5. PLAN DEL ESTUDIO, DEFINICIÓN DE ANÁLISIS INTERMEDIOS

Durante el transcurso del proyecto se realizarán un total de 8 visitas de estudio: visita de screening, visita basal, visita a la semana de iniciar tratamiento (excepto los pacientes que no inicien tratamiento), visita al mes 1, mes 3, mes 6, mes 9 y mes 12.

Después de aceptar la participación en el estudio y firmar el consentimiento informado los participantes serán citados para una nueva visita (visita de screening). En esa visita se llevará a cabo la valoración de los criterios de inclusión y de exclusión, y se realizará la evaluación neuropsicológica. En un plazo máximo de 3 meses se obtendrán los resultados y aquellos sujetos que cumplan criterios y que muestren alteración neurocognitiva serán incluidos definitivamente en el estudio. Serán citados para una nueva visita (visita basal), donde se randomizará a los sujetos a una de las 3 ramas de estudio y se les explicará los procedimientos específicos en cada grupo.

En la visita basal se recogerán las variables demográficas y se evaluarán las variables clínicas (excepto la variable de adhesión al tratamiento), emocionales, funcionales y de calidad de vida (excepto las escalas de satisfacción). Además se realizarán los analisis de laboratorio propios de la práctica clínica habitual.

A partir de la visita basal, a los pacientes que se hayan randomizado al grupo de litio, se les realizará el ajuste de dosis en cada una de las visitas. Tras hacerse la analítica, en caso de ser necesaria la modificación de la dosis, el paciente será contactado telefónicamente para informarle de los ajustes necesarios en el tratamiento. A los pacientes que hayan sido randomizados al grupo rivastigmina, será en la visita de la semana 1 cuando se les realice el ajuste de dosis.

En la visita de la semana 1 además de los ajustes de dosis, se recogerán los posibles acontecimientos adversos que se hayan podido producir y se realizará una nueva analítica para evaluar las posibles toxicidades.

En la visita del mes 1 se realizará la evaluación de las variables clínicas específicas para los grupos que hayan iniciado tratamiento, y de los posibles acontecimientos adversos que se hayan producido, además de la respectiva analítica.

Al mes 3 se llevarán a cabo exactamente las mismas evaluaciones que en la visita basal, y además se valorará la adhesión, las escalas de satisfacción, los acontecimientos adversos y se realizará una nueva exploración neuropsicologica. En la visita del mes 6 se recogerá la misma información a excepción de la exploración neuropsicologica. En la visita del mes 9 se recogerá la misma información que en el mes 1. Finalmente, en la visita del mes 12 se realizará exactamente la misma evaluación que en la visita del mes 3.

En cada control, además de la analítica de rutina, se guardará una muestra de plasma para la determinación del genotipo en caso de presentar alteraciones de la carga viral.

Está previsto realizar un análisis intermedio de eficacia cuando todos los pacientes hayan alcanzado el tercer mes de tratamiento. En cuanto a seguridad, se valorará en cada

visita individualmente, y al mes 3 esta valoración se hará también entre grupos de tratamiento.

5.6. TÉCNICA DE ENMASCARAMIENTO

No procede al tratarse de un ensayo clínico abierto.

5.7. CUADERNO DE RECOGIDA DE DATOS (CRD)

El contenido del Cuaderno de Recogida de Datos (CRD) se adjunta en el anexo I.

6.- SELECCIÓN DE SUJETOS

6.1. CRITERIOS PARA EL DIAGNÓSTICO DE LA PATOLOGÍA EN ESTUDIO

Los criterios diagnósticos se basarán en la historia clínica y exploración física.

6.2. CRITERIOS DE INCLUSIÓN

1. Edad entre 20 y 50 años.
2. Comprensión de los objetivos del estudio.
3. Firma del consentimiento informado (ver apartado Anexos).
4. Infección por VIH demostrada por prueba Western Blot o 2 pruebas Elisa.
5. Presencia de trastorno neurocognitivo asociado al VIH según clasificación diagnóstica de Antinori y cols (Neurology, 2007).
6. Estar en tratamiento antirretroviral.
7. Ser castellano o catalano parlante.

6.3. CRITERIOS DE EXCLUSIÓN

1. Estar en tratamiento antipsicótico, antidepresivo o ansiolítico.
2. Realizar tratamiento con otros fármacos que puedan interaccionar con los fármacos de estudio.
3. Estar en seguimiento psicoterapéutico.
4. Diagnóstico previo de infección oportunista con posible afectación del SNC.
5. Diagnóstico actual de trastorno psiquiátrico.
6. Diagnóstico de alteración neurológica con posible afectación del SNC.
7. Consumo actual de sustancias tóxicas.
8. Mujer en periodo de lactancia, embarazada o fértil con intención de quedarse embarazada.

6.4. NÚMERO DE SUJETOS

El ensayo se llevará a cabo en una muestra total de 45 pacientes (15 en el grupo con litio, 15 en el grupo con rivastigmina y 15 en el grupo control).

6.5. CRITERIOS DE RETIRADA Y ABANDONO

Los pacientes finalizarán el ensayo clínico antes del tiempo estipulado en las siguientes circunstancias:

- Aparición de acontecimientos adversos graves relacionados con la medicación en estudio.
- Proceso o enfermedad intercurrente que en opinión del investigador requiera la retirada del paciente.
- El paciente no desee continuar en el ensayo.

Si el estudio se tuviera que interrumpir prematuramente, todos los materiales (CRDs, etc.) deben ser devueltos por el investigador principal a la *Fundació Lluita Contra la SIDA.*

6.5.1. Actuación médica ante la retirada

En caso de producirse alguna retirada, se deberá cumplimentar **el formulario de fin de estudio** del CRD.

Se aportará información detallada sobre la fecha y motivos de la finalización al promotor. Es decir, como norma general, todos los pacientes que finalicen el tratamiento prematuramente serán sometidos a un examen clínico y a todas las pruebas que se especifican en esta visita.

El investigador facilitará el soporte médico necesario al paciente que finalice prematuramente el ensayo clínico por la aparición de un acontecimiento adverso o por la aparición de una enfermedad intercurrente.

6.6. PÉRDIDAS PREALEATORIZACIÓN

No se valorarán.

6.7. DURACIÓN APROXIMADA DEL PERIODO DE RECLUTAMIENTO

La duración aproximada del periodo de reclutamiento o inclusión será de 48 semanas.

7.- DESCRIPCIÓN DEL TRATAMIENTO

7.1. TRATAMIENTOS EXPERIMENTAL Y CONTROL

- Grupo litio: Pacientes que iniciarán tratamiento con litio.
- Grupo rivastigmina: Pacientes que iniciarán tratamiento con rivastigmina.
- Grupo control: Pacientes que no iniciarán tratamiento.

Se utilizará la forma comercial de todos los tratamientos.

7.2. SUMINISTRO, ACONDICIONAMIENTO, ETIQUETADO Y ALMACENAMIENTO

Los regímenes deberán ser suministrados por el servicio de Farmacia del centro.
Toda medicación para el ensayo clínico deberá ser almacenada en un lugar seguro mientras dure el mismo.

Litio
No requiere condiciones especiales de conservación.

Rivastigmina
No conservar a temperatura superior a 25°C.
Conservar el parche transdérmico dentro del sobre hasta su uso.

7.3. DOSIS, INTERVALO, VÍA Y FORMA DE ADMINISTRACIÓN

- Grupo litio: Pacientes que iniciarán tratamiento con litio, en formulación de comprimidos, inicialmente con una dosis de 400 mg. 2 veces al día y posteriormente ajustando la dosis según controles séricos de niveles de fármaco.

- Grupo rivastigmina: Pacientes que iniciarán tratamiento con rivastigmina, en formulación de parche transdérmico, iniciando con una dosis de 4,6 mg. una vez al día y posteriormente aumentando la dosis a 9,5 mg. una vez al día.

- Grupo control: Pacientes que no iniciarán tratamiento.

Específicamente, en los pacientes que inicien tratamiento con litio la dosis variará entre ellos, y se ajustará en función de la respuesta del sujeto y de los valores de litemia. Los niveles séricos de litio se determinarán en cada una de las visitas, con el objetivo de estabilizar los niveles de fármaco en sangre. Las dosis habituales están entre 1 y 4 comprimidos al día, lo que proporciona unas litemias de 0,7 y 1,2 mEq/l. La litemia mínima eficaz recomendada oscila de 0,5 a 0,8 mEq/l, y la máxima no debe sobrepasar los 2 mEq/l. En los pacientes que inicien tratamiento con rivastigmina se iniciará tratamiento con una dosis de 4,6 mg. una vez al día y en una semana se aumentará la dosis a 9,5 mg.

7.4. MODIFICACIÓN DE LA PAUTA DE TRATAMIENTO

No están previstos cambios en la pauta del tratamiento durante el estudio.

En caso de reacciones adversas a la medicación, el investigador retirará el fármaco, saliendo fuera del estudio.

7.5. TRATAMIENTOS CONCOMITANTES

Todos los tratamientos administrados durante el periodo de estudio, aparte de la medicación del ensayo clínico, se considerarán tratamientos concomitantes y deben documentarse en el CRD.

Se recuerda que los pacientes que participen en el estudio no deben seguir ningún tratamiento concomitante sin el conocimiento y el permiso del investigador.

**Medicaciones que no deben ser administradas durante el estudio por su interacción con el litio.**	
**Fármaco**	**Comentario clínico**
Diuréticos: diuréticos tiazídicos, furosemida, bumetanida	La administración concomitante de diuréticos y litio ejerce un efecto antidiurético paradójico, por lo que los diuréticos se deben utilizar con precaución en estos pacientes. Los diuréticos tiazídicos disminuyen el aclaramiento renal de litio con lo que aumentan los niveles séricos y pueden causar intoxicación por litio. En pacientes tratados de forma concomitante con tiazidas y litio deberá disminuirse la dosis de este último al objeto de mantener la litemia dentro de los niveles terapéuticos**.** Si bien, en caso de ser necesaria la administración concomitante de diuréticos y litio se recomienda disminuir la dosis de litio y utilizar preferentemente diuréticos de asa como furosemida y bumetanida
Psicofármacos: haloperidol, fluoxetina	Se ha descrito la aparición de un síndrome encefalopático seguido de lesión cerebral irreversible en casos aislados con la utilización conjunta de litio y haloperidol. El síndrome encefalopático se caracteriza por debilidad, letargia, fiebre, temblor y confusión, síntomas extrapiramidales, leucocitosis, aumento de enzimas hepáticos, BUN y glucosa. Aunque no se ha establecido una relación causal entre este síndrome y la administración concomitante de litio y haloperidol y la mayoría de los pacientes<br>reciben el tratamiento conjunto sin presentar efectos adversos, se recomienda una estrecha monitorización con objeto de poder retirar el tratamiento si aparecen signos de toxicidad neurológica. Existe la posibilidad de interacciones similares con otros fármacos antipsicóticos. El uso concomitante de litio y fluoxetina puede producir aumento o disminución de los niveles plasmáticos de litio y efectos adversos neuromusculares.
Antiinflamatorios no esteroideos: indometacina, ácido mefenámico,	Se ha descrito que aumentan los niveles plasmáticos de litio dando lugar a toxicidad por litio en algunos casos.

fenilbutazona, piroxicam y ibuprofeno	En caso de que se deban asociar AINEs y litio se recomienda una monitorización más frecuente de los niveles séricos de litio.
Anticonvulsivantes: carbamacepina y fenitoína.	Se ha descrito la aparición de efectos adversos neurológicos en pacientes que reciben conjuntamente estos fármacos.
Inhibidores del enzima de conversión de la angiotensina: captopril y enalapril	La administración concomitante de litio y de un inhibidor del enzima de conversión de la angiotensina debe realizarse con precaución ya que la pérdida de sodio puede disminuir el aclaramiento plasmático de litio, aumentar sus niveles plasmáticos con riesgo de intoxicación por litio. Su utilización conjunta puede requerir un reajuste de la dosis de litio en función de sus niveles séricos.
Antagonistas del calcio: verapamil	Las concentraciones plasmáticas de litio pueden disminuir tras iniciar tratamiento con verapamil en pacientes estabilizados de tratamiento con litio. En caso de requerirse la utilización conjunta, al introducir o al finalizar el tratamiento con verapamil, se realizará una estrecha monitorización del paciente y el reajuste de dosis de litio, si procede. Los agentes antagonistas del calcio pueden asimismo potenciar los efectos tóxicos del litio (neurotóxicos, gastrointestinales y bradicardia).
Agentes bloqueantes neuromusculares: succinilcolina y pancuronio	El litio prolonga el tiempo de bloqueo neuromuscular inducido por la succinilcolina y el pancuronio. Se recomienda interrumpir temporalmente el tratamiento con litio 48-72 horas antes de la utilización de estos medicamentos.
Ioduros	El uso concomitante de litio y ioduros puede resultar en un efecto hipotiroideo aditivo o sinérgico.
Sodio	El aclaramiento renal de litio puede aumentar o disminuir de un 30 a un 50% con el aumento o la disminución de la ingesta de sodio, respectivamente. Deberá advertirse a los pacientes que eviten cambios importantes en la ingesta de sodio.
Otros	Los siguientes fármacos pueden disminuir los niveles séricos de litio por aumento de su excreción urinaria: acetazolamida, urea, xantinas, agentes alcalinizantes tales como el bicarbonato sódico. Pueden necesitarse dosis superiores de litio en pacientes que reciban estos fármacos concomitantemente.

No se han realizado estudios de interacciones específicos con rivastigmina en parches transdérmicos.
Como inhibidor de las colinesterasas, la rivastigmina puede potenciar excesivamente los efectos de los relajantes musculares del tipo succinilcolina durante la anestesia. Se recomienda seleccionar cuidadosamente los agentes anestésicos. Si es necesario, debe considerarse un ajuste de dosis o una interrupción temporal del tratamiento.
Teniendo en cuenta sus efectos farmacodinámicos, la rivastigmina no debe administrarse junto con otras sustancias colinérgicas, y además podría interferir con la actividad de medicamentos anticolinérgicos.

7.6. CUMPLIMIENTO

En ambos grupos se controlará la adhesión a los tratamientos de estudio y al tratamiento antirretroviral en cada una de las visitas. Esto se evaluará a través de la aplicación del cuestionario Self-Reported Adherence (SERAD), previamente adaptado y validado en población española infectada por el VIH.[40] Concretamente se usará una versión reducida de este instrumento, la cual contemplará un índice de adhesión al tratamiento en el último mes y en la última semana. Estos índices se calcularán en función del número de dosis omitidas respecto al número de dosis prescritas.

7.7. NORMAS PARA EL MANEJO DE LOS TRATAMIENTOS DE ESTUDIO

Litio
No requiere condiciones especiales de conservación.

Rivastigmina
No conservar a temperatura superior a 25°C.
Conservar el parche transdérmico dentro del sobre hasta su uso.

8.- DESARROLLO DEL ENSAYO Y EVALUACIÓN DE LA RESPUESTA

8.1. CRITERIOS PARA LA EVALUACIÓN DE LA RESPUESTA

8.1.1. VARIABLE PRINCIPAL A ESTUDIAR

Serán 2 las variables principales de valoración: el porcentaje de pacientes con alteración neurocognitiva y el cambio en un índice global de alteración. Para ello se evaluarán las siguientes variables neurocognitivas: Se evaluarán 7 áreas, incluyendo la atención/memoria de trabajo, velocidad en el procesamiento de la información, memoria verbal, aprendizaje, funciones ejecutivas, fluencia verbal, y función motora. La inteligencia premórbida también será evaluada, aunque tratada como variable control entre grupos. Estas variables neurocognitivas serán medidas gracias a la pasación de una batería de pruebas neuropsicológicas. Dicha batería será compuesta en función de las recomendaciones internacionales para la evaluación del funcionamiento neurocognitivo en los pacientes con VIH, y será aplicada por un neuropsicológo formado específicamente en la aplicación de estas pruebas. El explorador no será consciente en ningún momento del grupo al que pertenecerá el sujeto, de manera que el estudio será considerado ciego a este nivel.

8.1.2.VARIABLES SECUNDARIAS A ESTUDIAR

- Variables demográficas y clínicas: edad, sexo, escolarización, ocupación, recuento de CD4+ nadir, carga viral máxima, años desde el diagnóstico de VIH, vía de transmisión, presencia de TARV, tipo de TARV, años en TARV y coinfección VIH/VHC, adhesión al tratamiento.

- Acontecimientos adversos asociados al inicio del tratamiento con litio y con rivastigmina y parámetros de toxicidad (Hematología: Hematocrito, Hematíes, Hemoglobina, VCM, Linfocitos, Plaquetas, Índice de Quick. Bioquímica: Glucosa, Urea, Creatinina, sodio, potasio, Bilirrubina, Proteínas totales, Albúmina, SGPT (ALAT), SGOT (ASAT), Gamma-GT, fosfatasa alcalina, Triglicéridos, Colesterol, HDL, LDL, Creatinin quinasa, Lactato, Tirotropina (TSH), tiroxina libre (T4L))

- Variables emocionales: Se evaluarán síntomas de depresión y de ansiedad gracias a la pasación del cuestionario HADS, recomendado particularmente para su uso en ámbito hospitalario.

- Variables funcionales: Se medirán 6 escalas de interferencia funcional respecto a actividades de la vida diaria, así como la evaluación autorreferida del funcionamiento neurocognitivo. Las primeras serán medidas gracias a la pasación del cuestionario IADL, y la autoevaluación neurocognitiva a través del cuestionario PAOFI.

- Variables de calidad de vida: Se cubrirán 4 escalas de calidad de vida obtenidas por el cuestionario MOS-HIV, y escalas de satisfacción tipo Likert basadas en los tratamientos que reciban.

8.1.3. ENDPOINTS DEL ESTUDIO

1. Porcentaje de pacientes con alteración neurocognitiva.
2. Déficit global de empeoramiento neurocognitivo.
3. Acontecimientos adversos asociados al inicio de la nueva medicación.
4. Porcentaje de pacientes que refieren queja cognitiva.
5. Medidas de las variables funcionales.
6. Puntuaciones en las variables de calidad de vida.
7. Puntuaciones en las escalas de satisfacción con respecto a los tratamientos de estudio.

8.2. PROCEDIMIENTOS DE LOS PARÁMETROS A EVALUAR

8.2.1. Historia clínica y variables demográficas

Se recogerán aquellos datos demográficos o relacionados con la infección por el VIH de interés para caracterizar a la población incluida en el esudio (edad, sexo, escolarización, ocupación, recuento de CD4+ nadir, carga viral máxima, años desde el diagnóstico de VIH, vía de transmisión, presencia de TARV, tipo de TARV, años en TARV y coinfección VIH/VHC.)

Relacionadas con la seguridad de los nuevos tratamientos, se valorarán en los 3 grupos de estudio los acontecimientos adversos asociados al inicio de la medicación.. Adicionalmente, en el grupo de tratamiento con litio se medirá periódicamente la concentración sérica de litio, la función renal y tiroidea. En ambos grupos será controlada la adhesión a los tratamientos de estudio. Estas variables serán recogidas por el clínico responsable (miembro del equipo investigador) según la información incluida en las historias clínicas de la Unidad de VIH o conseguida tal y como se desarrolle en la práctica clínica habitual de la asistencia a pacientes con VIH.

8.2.2. Análisis de laboratorio

Se obtendrán muestras de sangre, tras al menos 8 horas de ayuno, en cada uno de los puntos especificados en el cronograma del estudio (apartado 8.3). Se cuantificarán los siguientes parámetros, según proceda:

- **Hematología:**
  - Hematocrito
  - Hematíes
  - Hemoglobina
  - VCM
  - Linfocitos
  - Plaquetas
  - Índice de Quick

- **Bioquímica:**
  - Glucosa
  - Urea
  - Creatinina
  - Ionograma: sodio, potasio
  - Bilirrubina
  - Proteínas totales
  - Albúmina
  - Transaminasas: SGPT (ALAT), SGOT (ASAT)
  - Gamma-GT, fosfatasa alcalina
  - Triglicéridos
  - Colesterol, HDL, LDL
  - Creatinin quinasa
  - Lactato
  - Tirotropina (TSH), tiroxina libre (T4L)

- **Concentraciones plasmáticas de litio**

- **Recuento actual de CD4+**
- **Carga viral actual**
- **Análisis de orina (tira de papel indicador)**
  Prueba de embarazo (si procede)

Nota: Antes de iniciar el estudio, el laboratorio facilitará al promotor y al investigador una lista de los valores normales para los parámetros de laboratorio a evaluar durante el estudio.

8.3. DESARROLLO DEL ENSAYO

El seguimiento de un caso durará 48 semanas y comprende 8 visitas al paciente en estudio: una de screening, una basal/aleatorización y 6 visitas de seguimiento. Todas las visitas son obligatorias, y además, el paciente debe asistir dentro de los límites establecidos: ±1 semana.

En cada visita, se seguirá el procedimiento que se indica en el siguiente cronograma.

Cronograma:

Tareas	Visitas									
	Screening			Basal	Sem 1[5]	Mes 1	Mes 3	Mes 6	Mes 9	Mes 12
	-3	-2	-1	0	1		3	6	9	12
Criterios de Inclusión / Exclusión	x									
Aceptación del Consentimiento Informado	x									
Aleatorización				x						
Exploración Neuropsicológica	x						x			x
Extracción / evaluación de parámetros analíticos (Bioquímica[1], Hematología)				x[2]	x	x	x	x	x	x
Prueba de Diagnóstico de Embarazo	x									
Evaluación de Acontecimientos Adversos					x	x	x	x	x	x
Cambio / Ajuste de Dosis (litio)					x	x	x	x	x	x
Cambio / Ajuste de Dosis (rivastigmina)					x					
Evaluación de las Variables Demográficas				x						
Evaluación de las Variables Clínicas				x[3]		x	x	x	x	x
Evaluación del Estado Emocional				x			x	x		x
Evaluación del Estado Funcional				x			x	x		x
Evaluación de la Calidad de Vida				x[4]			x	x		x
Almacenaje de plasma				x		x	x	x	x	x

[1] La evaluación de las hormonas tirotropina (TSH) y tiroxina libre (T4L) solo se realizará en aquellos pacientes que hayan iniciado tratamiento con litio.
[2] En esta analítica no será necesaria la evaluación de los niveles de litio.
[3] Excepto la variable de adhesión al tratamiento.
[4] Excepto las escalas de satisfacción.
[5] Únicamente la realizarán aquellos pacientes que hayan iniciado tratamiento.

9.- ACONTECIMIENTOS ADVERSOS

9.1. DEFINICIÓN

Acontecimiento adverso (AA): Cualquier suceso médico desfavorable que pueda presentar un paciente o sujeto de investigación clínica al que se ha administrado un producto farmacéutico y que no tiene necesariamente que tener una relación causal con dicho tratamiento.

Acontecimiento adverso grave (AAG): Cualquier AA que, independientemente de la dosis:

- Produce la muerte.
- Pone en peligro la vida.
- Precisa de ingreso hospitalario o lo prolonga.
- Produce una discapacidad/incapacidad persistente o importante, así como
- una anomalía o defecto congénito.
- Es un suceso no incluido en los párrafos anteriores pero que puedan poner en peligro al paciente o puedan requerir intervención para prevenir cualquiera de los resultados anteriormente mencionados.

Acontecimiento adverso inesperado: es aquel AA relacionado con el tratamiento en estudio, cuya naturaleza o intensidad no coincide con la información disponible del producto (Ficha técnica, para productos comercializados).

9.2. DESCRIPCIÓN DE LOS CRITERIOS DE IMPUTABILIDAD

La relación de causalidad se establecerá según el algoritmo del Sistema Español de Farmacovigilancia, en la que se definen las siguientes categorías:

Definitiva:

- Existe una secuencia temporal plausible en relación con la administración del fármaco o con los niveles plasmáticos o tisulares del mismo.
- La manifestación observada coincide con el esquema de reacciones adversas conocidas del fármaco implicado.
- No puede ser explicado por la enfermedad concurrente o por otros fármacos o sustancias químicas.
- La respuesta a la retirada debe ser clínicamente plausible. O sea mejora al interrumpir la administración del fármaco.
- El proceso de reexposición debe ser positivo.

Probable:

- Existe una secuencia temporal razonable a la exposición del fármaco.
- La manifestación observada coincide con el esquema de reacciones adversas conocidas del fármaco implicado.
- Es poco probable que pueda atribuirse a la enfermedad intercurrente o a otros fármacos o sustancias químicas.
- Después de ser retirado el fármaco sigue una secuencia clínica razonable.
- Mejora al interrumpirse la administración del fármaco.
- No se requiere reexposición para completar esta definición.

Posible:

- Existe una secuencia temporal razonable a la exposición del fármaco.

- Coincide con el esquema de reacciones adversas conocidas.
- Puede ser debida a la situación clínica del paciente o a otros fármacos o sustancias químicas administrados concomitantemente.
- La información sobre la retirada puede no existir o ser confusa.

Improbable:

- Un acontecimiento clínico, incluidas anomalías en las pruebas de laboratorio, con una relación temporal respecto a la administración del fármaco que hace improbable la relación de causalidad, y en el que otros fármacos, sustancias químicas o enfermedad intercurrente proporcionan explicaciones plausibles.

No relacionado:

No cumple ninguno de los criterios mencionados.

9.3. PROCEDIMIENTO PARA LA NOTIFICACIÓN DE LOS ACONTECIMIENTOS ADVERSOS

El investigador:

El investigador notificará de forma inmediata al promotor los **acontecimientos adversos graves (AAG)** (ver apartado 9.1.)

La notificación se llevará a cabo en las primeras 24 horas tras su aparición, mediante la cumplimentación del formulario de notificación de acontecimientos adversos graves (anexo V de este protocolo), que se enviará al promotor por fax al núm. 93 465 76 02.

Se registrarán todos los acontecimientos adversos independientemente de la relación de causalidad imputada, en el formulario de descripción de acontecimientos adversos. Este formulario se encuentra en el CRD de cada participante en el estudio (ver anexo I).

Dependiendo de la naturaleza del acontecimiento adverso a evaluar se clasifica en:

- grave / no grave
- inesperada / esperada

Las características del acontecimiento, gravedad y "esperabilidad" definen cuándo y cómo se debe notificar esta información a las autoridades sanitarias.

La recogida de acontecimientos adversos deberá llevarla a cabo el equipo investigador del ensayo, se especificarán indicando el momento de aparición expresado en la mínima unidad temporal posible, si es grave o no grave y si es esperado o no esperado, la intensidad del mismo (leve, moderada o severa), las medidas adoptadas (ninguna, tratamiento, interrupción del tratamiento del estudio), la evolución (remisión total, remisión parcial, persiste) y la imputabilidad en la que se utilizarán los criterios que se definen en el apartado 9.2.

La intensidad será determinada por el investigador utilizando los siguientes términos según los criterios establecidos por la OMS:

- Grado I-II
- Grado III-IV

El promotor:

El promotor notificará a la AEMPS, a los órganos competentes de la CCAA donde se realice el ensayo y a los CEICs implicados cualquier información importante de seguridad del medicamento en investigación.

El promotor notificará a la AEMPS los acontecimientos adversos graves e inesperados asociados a medicamento en investigación, cuando el medicamento no se encuentre comercializado. Si el medicamento está comercializado se comunicará siguiendo los requerimientos de farmacovigilancia.

El promotor notificará a los órganos competentes de les CCAA, en cuyo territorio se este realizando el ensayo, de forma individual, y a los CEICs implicados, los acontecimientos adversos graves e inesperados asociados a medicamento en investigación, y que hayan ocurrido en pacientes seleccionados en sus respectivos ámbitos territoriales.

A partir del momento que el promotor haya tenido conocimiento de la sospecha de reacción adversa, el plazo de notificación será de:

- 15 días naturales
- 7 días si el acontecimiento adverso grave e inesperado haya ocasionado la muerte o puesto en peligro la vida del sujeto. Más 8 días para completar toda la información.

Si la comunicación se realiza en formato informático, no será necesario la notificación a las CCAA.

El promotor mantendrá un registro detallado de todos los acontecimientos adversos comunicados por los investigadores.

Todos los acontecimientos adversos serán notificados de forma tabulada en el informe final del estudio.

10.- ASPECTOS ÉTICOS

10.1. CONSIDERACIONES GENERALES

10.1.1. Declaración de Helsinki

El ensayo clínico se llevará a cabo de acuerdo con los principios de la Declaración de Helsinki (última revisión).

10.1.2. Comités éticos y autoridad reguladora

Este estudio se realizará según la reglamentación española y la documentación requerida previa a su inicio será:

- Aceptación del protocolo por parte del investigador y el promotor.
- Dictamen único del CEIC de referencia.
- Autorización de la Agencia Española del Medicamento y Productos Sanitarios.

10.2. INFORMACIÓN AL PACIENTE Y CONSENTIMIENTO INFORMADO

El consentimiento informado (Anexo III) se obtendrá antes de incluir al paciente en el estudio. El investigador deberá informar al paciente de la naturaleza, duración y propósito de este estudio y, además, de todos los inconvenientes y obstáculos que, dentro de lo razonable, pueden esperarse. Además se proporcionará información por escrito al paciente. Los pacientes participantes deben tener capacidad legal para dar su consentimiento y ejercer su libertad de decisión. El paciente tiene derecho a retirarse del estudio en cualquier momento.

10.3. CONFIDENCIALIDAD. PROTECCIÓN DEL PACIENTE

El procesamiento de los datos que el Promotor del Estudio recopile durante el estudio estará sujeto a la legalidad vigente en cuanto a la protección de datos (LOPD, Ley Orgánica 15/1999, de 13 de diciembre de protección de datos de carácter personal). Los pacientes se identificarán en los registros únicamente con el número de código. Al paciente se le deberá garantizar el anonimato y se le deberá informar que toda comunicación se llevará a cabo entre él y el investigador, y no con el promotor del estudio.

11.- CONSIDERACIONES PRÁCTICAS

11.1. RESPONSABILIDADES DE LOS PARTICIPANTES EN EL ESTUDIO

Obligaciones del Investigador
Los investigadores deberán llevar a cabo el estudio de acuerdo con el diseño establecido y siguiendo todas las directrices del presente protocolo y de las enmiendas incorporadas a éste.

Personal auxiliar
El personal auxiliar que colabore en el estudio, deberá seguir las normas establecidas y las órdenes de los investigadores. Así mismo deberá comunicar cualquier eventualidad a los investigadores.

Normas para los pacientes
Los pacientes serán advertidos e instruidos para que cumplan con las recomendaciones que hagan posible el desarrollo del estudio, tal como se contempla en este protocolo.

11.2. MODIFICACIONES DEL PROTOCOLO

Las modificaciones, enmiendas o ampliaciones al protocolo, irán firmadas por el Investigador Principal y promotor y se comunicarán al Comité Ético de Investigación Clínica de referencia, a los CEIC implicados y a la Agencia Española del Medicamento y Productos Sanitarios.

En caso de modificación relevante del protocolo se requerirá el informe previo del Comité Ético de Investigación Clínica y la autorización de la Agencia Española del Medicamento y Productos Sanitarios.

11.3. RECOGIDA Y ARCHIVO DE DATOS

La recogida y trascripción de los datos al CRD será realizada por el investigador y colaboradores, que cumplimentarán el CRD de acuerdo con los procedimientos normalizados de trabajo, conociendo y respetando el protocolo del estudio.

El dato original deberá ser legible y escrito con bolígrafo negro o azul. No será aceptable escribir con lápiz o pluma. Cualquier corrección en el CRD deberá realizarse tachando con una raya los datos incorrectos y dejando que los datos correctos resulten claramente visibles. No se borrarán los datos incorrectos. Las correcciones irán firmadas y fechadas.

En aquellos centros dónde sea posible, la recogida de datos se realizará directamente en formato electrónico.

La información recogida en el CRD será considerada como dato primario, a excepción de los datos de filiación del paciente. Debe recogerse en la historia clínica la participación del paciente en el estudio, así como el número de código asignado y la identificación de las diferentes visitas que tendrán lugar a lo largo del estudio.

Los datos serán introducidos en una base de datos electrónica para su posterior análisis estadístico.

El archivo de datos se ajustará a los procedimientos del promotor.

La Lista de Identificación de Sujetos y el consentimiento informado se guardarán aparte en el archivo del investigador en el centro correspondiente. Estos documentos se guardarán un mínimo de 5 años.

11.4. BUENA PRÁCTICA CLÍNICA

Este estudio se adecuará a las normas de "Buena Práctica Clínica".

Los investigadores e instituciones permitirán las monitorizaciones y auditorias por parte de las autoridades sanitarias o el promotor dando acceso directo a los datos y documentos fuente originales.

11.5 PUBLICACIÓN

La publicación de los resultados del ensayo cumplirá los requisitos que se establecen en el artículo 38 del Real Decreto 223/2004.

11.6. DOCUMENTACIÓN DEL ESTUDIO

1. Protocolo.
2. ANEXO I: CUADERNO DE RECOGIDA DE DATOS
3. ANEXO II: MANUAL DEL INVESTIGADOR
4. ANEXO III: INFORMACIÓN AL PACIENTE Y CONSENTIMIENTO POR ESCRITO
5. ANEXO IV: SEGURO DE RESPONSABILIDAD CIVIL
6. ANEXO V: FORMULARIO DE NOTIFICACIÓN DE ACONTECIMIENTOS ADVERSOS GRAVES

12.- ANÁLISIS ESTADÍSTICO

Se creará una base de datos informatizada que se empleará para la introducción y gestión de los datos. Esta fase de introducción de datos se realizará simultáneamente al seguimiento clínico de los pacientes. Todos los análisis estadísticos se llevarán a cabo mediante el paquete estadístico SPSS 15.0.

El tratamiento de los datos de carácter personal de los sujetos participantes en este proyecto se ajustará a la Ley Orgánica 15/1999 de 13 de Diciembre de Protección de Datos de carácter personal y a las demás normas concordantes y complementarias en la materia, por lo que tanto el investigador principal del proyecto, como los investigadores participantes, adoptarán las medidas de índole técnica y organizativa necesarias que garanticen la seguridad de estos datos y eviten su alteración, pérdida, tratamiento o acceso no autorizado.

12.1. POBLACIONES PARA EL ANÁLISIS

Se emplearán dos poblaciones para el análisis de la eficacia y seguridad: la población "por-protocolo" o de casos válidos y la población "por intención de tratar" o de casos aleatorizados.

Se define la población "por intención de tratar" (ITT) como el conjunto de todos los pacientes aleatorizados, que hayan recibido medicación a estudio y de los cuales se disponga, por lo menos, de una medición de la eficacia.

La población "por protocolo" (PP) consistirá en un sub-grupo de la población ITT, definido en base al cumplimiento de los criterios de inclusión y exclusión, de los criterios de retirada y abandono y de los procedimientos descritos en el protocolo.
El análisis principal de eficacia se realizará en la población PP.

El análisis de seguridad incluirá todos los pacientes que hayan recibido medicación a estudio.

Se listarán todos los pacientes seleccionados para el estudio que hayan sido excluidos de cada una de las poblaciones arriba mencionadas, tabulándose de forma descriptiva las características que de ellos se disponga.

12.2. DESCRIPCIÓN DE LA MUESTRA

Se realizará una descripción de la muestra incluyendo los siguientes análisis:
Análisis descriptivo general de todas las variables del estudio y lo mismo separando por grupos de tratamiento. Para ello se emplearán análisis descriptivos de estimación de medias, desviación estándar, mediana, rango intercuartílico, valor máximo y valor mínimo para las variables cuantitativas y para las variables categóricas las frecuencias absolutas y relativas de cada categoría.

Se verificará la comparabilidad de los grupos de tratamiento en cuanto a sus características basales (variables medidas hasta el momento de la toma de la medicación). En caso de observarse que los grupos de tratamiento están desbalanceados respecto a alguna característica que se considere de posible influencia sobre la valoración de la eficacia, se evaluará la realización de un ajuste por tales factores en el análisis de las variables de eficacia.

12.3. ANÁLISIS DE EFICACIA

Para el análisis de eficacia se considerarán 2 variables principales de valoración, ambas recogidas durante el período de seguimiento: el porcentaje de pacientes con alteración neurocognitiva y un índice global de alteración. La alteración neurocognitiva será determinada tal y como ha sido sugerido en la clasificación diagnóstica propuesta por Antinori y cols (Neurology, 2007). El índice global de alteración estará basado en el índice GDS, un método validado que calcula un score integrado de la medición de las 7 áreas neurocognitivas a evaluar, y que además se basa en la estandarización de las puntuaciones por edad, escolarización y sexo.

El análisis principal se basará en la comparación de los grupos de tratamiento, entre ellos y con respecto al grupo control. Además se considerará la diferencia intra grupo de las medidas de eficacia al inicio del estudio (visita basal), en el análisis preliminar (visita mes 3), y al final del estudio (visita mes 12). Para las comparaciones se utilizarán pruebas de comparación de medias (t-test), medianas (pruebas no paramétricas) o proporciones (Chi-cuadrado, Test de Fisher o McNemar), según el tipo de variable, su distribución, y si los datos son apareados o no.
De la misma manera se describirá y comparará la satisfacción respecto el tratamiento, la mejora funcional y la calidad de vida en los grupos de estudio.Se considerará emplear transformaciones de los datos.

Se valoraran las posibles causas de datos perdidos, de manera que si hubiera indicios de observaciones perdidas no al azar, éstas se tabularán y describirán. Si por el contrario, los datos perdidos son al azar o completamente al azar se seguirá con el plan de análisis descrito.

12.4. ANÁLISIS DE SEGURIDAD

El análisis de seguridad se basará en la tolerabilidad a los tratamientos, definida a partir de los acontecimientos adversos aparecidos. Esta información será analizada de forma descriptiva gráfica y/o tabular. Se harán comparaciones de la frecuencia de efectos adversos de los tratamientos, entre ellos y comparándolos con el grupo control. Se evaluará la relevancia clínica de los valores, así como los cambios sucedidos entre distintos controles.

12.5. DESCRIPCIÓN DEL TAMAÑO DE LA MUESTRA

El número de sujetos experimentales propuesto para el estudio es de 15 individuos por rama (un total de 45 participantes). Este tamaño muestral ha sido calculado estadísticamente y posteriormente comprobado en función de la potencia asistencial y de inclusión de participantes en ensayos clínicos de la unidad donde se realizará la investigación. Si se incluye un total de 45 unidades experimentales, distribuidas entre los 3 grupos según el ratio 1:1:1, se obtendrá una potencia del 74,2% para detectar diferencias en el contraste de la hipótesis nula, la cual considera que las proporciones de los 3 grupos serán iguales, contra la hipótesis alternativa, la cual considera no igualdad mediante una prueba Chi cuadrado para 3 muestras independientes, teniendo en cuenta que el nivel de significación es del 5%, y asumiendo que los porcentajes esperados de respuesta los 3 grupos son, aproximadamente, de 75% en la rama de litio, 75% en la de rivastigmina, y 15% en el grupo control.

13. REFERENCIAS

1. Heaton RK, Grant I, Butters N, et al. The HNRC 500--neuropsychology of HIV infection at different disease stages. HIV Neurobehavioral Research Center. J Int Neuropsychol Soc. 1995 May;1(3):231-51.
2. Reger M, Welsh R, Razani J, et al. A meta-analysis of the neuropsychological sequelae of HIV infection. J Int Neuropsychol Soc. 2002 Mar;8(3):410-24.
3. Woods SP, Moore DJ, Weber E, et al. Cognitive neuropsychology of HIV-associated neurocognitive disorders. Neuropsychol Rev. 2009 Jun;19(2):152-68.
4. Clifford DB. HIV-associated neurocognitive disease continues in the antiretroviral era. Top HIV Med. 2008 Jun-Jul;16(2):94-8.
5. Antinori A, Arendt G, Becker JT, et al. Updated research nosology for HIV-associated neurocognitive disorders. Neurology. 2007 Oct 30;69(18):1789-99.
6. Tozzi V, Balestra P, Galgani S, et al. Neurocognitive performance and quality of life in patients with HIV infection. AIDS Res Hum Retrov 2003; 19: 643-652.
7. Heaton RK, Marcotte TD, Mindt MR, et al; HNRC Group. The impact of HIV-associated neuropsychological impairment on everyday functioning. J Int Neuropsychol Soc 2004; 10: 317-331.
8. Gorman AA, Foley JM, Ettenhofer ML, et al. Neuropsychol Rev. 2009 Jun;19(2):186-203. Functional consequences of HIV-associated neuropsychological impairment. Epub 2009 May 27.
9. Tozzi V, Balestra P, Serraino D, et al. Neurocognitive impairment and survival in a cohort of HIV-infected patients treated with HAART. AIDS Res Hum Retroviruses 2005; 21:706-13.
10. Ellis RJ, Deutsch R, Heaton RK, et al. Neurocognitive impairment is an independent risk factor for death in HIV infection. San Diego HIV Neurobehavioral Research Center Group. Arch Neurol 1997; 54: 416-24.
11. Jevtović Dj, Vanovac V, Veselinović M, et al. The incidence of and risk factors for HIV-associated cognitive-motor complex among patients on HAART. Biomed Pharmacother. 2009 Sep;63(8):561-5.
12. Bhaskaran K, Mussini C, Antinori A, et al; CASCADE Collaboration. Changes in the incidence and predictors of human immunodeficiency virus-associated dementia in the era of highly active antiretroviral therapy. Ann Neurol. 2008 Feb;63(2):213-21.
13. Valcour V, Yee P, Williams AE, et al. Lowest ever CD4 lymphocyte count (CD4 nadir) as a predictor of current cognitive and neurological status in human immunodeficiency virus type 1 infection--The Hawaii Aging with HIV Cohort. J Neurovirol. 2006 Oct;12(5):387-91.
14. Robertson KR, Smurzynski M, Parsons TD, et al. The prevalence and incidence of neurocognitive impairment in the HAART era. AIDS. 2007 Sep 12;21(14):1915-21.
15. Clifford DB, Yang Y, Evans S. Neurologic consequences of hepatitis C and human immunodeficiency virus coinfection. J Neurovirol. 2005;11 Suppl 3:67-71.
16. Letendre SL, Ellis RJ, Everall I, et al. Neurologic complications of HIV disease and their treatment. Top HIV Med. 2009 Apr-May;17(2):46-56.
17. Muñoz-Moreno JA, Letendre S, McClernon D, et al. Persistent HIV in the Central Nervous System during Treatment is Associated with Worse ART Penetration and Cognitive Impairment. The 9th International Symposium on Neurovirology. 2-6 June, 2009, Miami, FL. Abstract P130.
18. Kaul M. HIV-1 associated dementia: update on pathological mechanisms and therapeutic approaches.Curr Opin Neurol. 2009 Jun;22(3):315-20.
19. Jayadev S, Garden GA. Host and viral factors influencing the pathogenesis of HIV-associated neurocognitive disorders. J Neuroimmune Pharmacol. 2009 Jun;4(2):175-89.
20. Ellis RJ, Calero P, Stockin MD. HIV infection and the central nervous system: a primer. Neuropsychol Rev. 2009 Jun;19(2):144-51.
21. Tozzi V, Balestra P, Galgani S, et al. Positive and sustained effects of highly active antiretroviral therapy on HIV-1-associated neurocognitive impairment.1999 Oct 1;13(14):1889-97.
22. Tozzi V, Balestra P, Galgani S, et al. Changes in neurocognitive performance in a cohort of patients treated with HAART for 3 years. J Acquir Immune Defic Syndr. 2001 Sep 1;28(1):19-27.
23. Simioni S, Cavassini M, Annoni JM, et al. Cognitive dysfunction in HIV patients despite long-standing suppression of viremia. AIDS. 2009 Dec 7.
24. Muñoz-Moreno JA, Fumaz CR, Ferrer MJ, et al. Nadir CD4 cell count predicts neurocognitive impairment in HIV-infected patients. AIDS Res Hum Retroviruses. 2008 Oct;24(10):1301-7.

25. Muñoz-Moreno JA, Pérez-Álvarez N, Letendre S, et al. Predicting HIV-related Neurocognitive Dysfunction: the Relevance of Clinical Factors. The 17th Conference on Retroviruses and Opportunistic Infections. 16-19 February 2009, San Francisco, CA. Abstract 416.
26. Tozzi V, Balestra P, Bellagamba R, et al. Persistence of neuropsychologic deficits despite long-term highly active antiretroviral therapy in patients with HIV-related neurocognitive impairment: prevalence and risk factors. J Acquir Immune Defic Syndr. 2007 Jun 1;45(2):174-82.
27. Muñoz-Moreno JA, Fumaz C, Prats A, et al. Interruptions of Antiretroviral Therapy in HIV Infection: Are They Detrimental to Neurocognitive Functioning? J Neurov [in press].
28. Tozzi V, Balestra P, Salvatori MF, et al. Changes in cognition during antiretroviral therapy: comparison of 2 different ranking systems to measure antiretroviral drug efficacy on HIV-associated neurocognitive disorders. J Acquir Immune Defic Syndr. 2009 Sep 1;52(1):56-63.
29. Schifitto G, Peterson DR, Zhong J, et al.Valproic acid adjunctive therapy for HIV-associated cognitive impairment: a first report. Neurology. 2006 Mar 28;66(6):919-21. Epub 2006 Mar 1.
30. Schifitto G, Yiannoutsos CT, Ernst T, et al; ACTG 5114 Team.Selegiline and oxidative stress in HIV-associated cognitive impairment. Neurology. 2009 Dec 8;73(23):1975-81.
31. Schifitto G, Navia BA, Yiannoutsos CT, et al; Adult AIDS Clinical Trial Group (ACTG) 301; 700 Teams; HIV MRS Consortium. Memantine and HIV-associated cognitive impairment: a neuropsychological and proton magnetic resonance spectroscopy study.AIDS. 2007 Sep 12;21(14):1877-86.
32. Schifitto G, Zhong J, Gill D, et al. Lithium therapy for human immunodeficiency virus type 1-associated neurocognitive impairment. J Neurovirol. 2009 Apr;15(2):176-86.
33. Letendre SL, Woods SP, Ellis RJ, et al; HNRC Group. Lithium improves HIV-associated neurocognitive impairment. AIDS. 2006 Sep 11;20(14):1885-8.
34. Birks J, Grimley Evans J, et al. Rivastigmine for Alzheimer's disease. Cochrane Database Syst Rev. 2009 Apr 15;(2):CD001191.
35. Darreh-Shori T, Jelic V. Safety and tolerability of transdermal and oral rivastigmine in Alzheimer's disease and Parkinson's disease dementia. Expert Opin Drug Saf. 2010 Jan;9(1):167-76
36. Ribeiz SR, Bassitt DP, Arrais JA, et al. Cholinesterase inhibitors as adjunctive therapy in patients with schizophrenia and schizoaffective disorder: a review and meta-analysis of the literature. CNS Drugs. 2010 Apr 1;24(4):303-17.
37. Krupp LB, Christodoulou C, Melville P, et al. Donepezil improved memory in multiple sclerosis in a randomized clinical trial. Neurology. 2004 Nov 9;63(9):1579-85.
38. Christodoulou C, Melville P, Scherl WF, Macallister WS, Elkins LE, Krupp LB. Effects of donepezil on memory and cognition in multiple sclerosis. J Neurol Sci. 2006 Jun 15;245(1-2):127-36.
39. Chitnis S, Rao J. Rivastigmine in Parkinson's disease dementia. Expert Opin Drug Metab Toxicol. 2009 Aug;5(8):941-55.
40. Muñoz-Moreno JA, Fumaz CR, Ferrer MJ, Tuldra A, Rovira T, Viladrich C, Bayes R, Burger DM, Negredo E, Clotet B; the SERAD Validation Team. Assessing Self-Reported Adherence to HIV Therapy by Questionnaire: The SERAD (Self-Reported Adherence) Study. AIDS Res Hum Retroviruses 2007 Oct; 23(10): 1166-1175.

ANEXO I: CUADERNO DE RECOGIDA DE DATOS (CRD)
(Adjunto)

ANEXO II: MANUAL DEL INVESTIGADOR
(Adjunto)

ANEXO III: HOJA DE INFORMACIÓN AL PACIENTE Y CONSENTIMIENTO INFORMADO
(Adjunto)

ANEXO IV: SEGURO
(Adjunto)

ANEXO V: FORMULARIO DE NOTIFICACIÓN DE SAE
(Adjunto)
